# Fast reconstruction and optical-sectioning three-dimensional structured illumination microscopy

**DOI:** 10.1016/j.xinn.2025.100812

**Published:** 2025-02-06

**Authors:** Ruijie Cao, Yaning Li, Wenyi Wang, Yunzhe Fu, Xiaoyu Bu, Dilizhatai Saimi, Jing Sun, Xichuan Ge, Shan Jiang, Yuru Pei, Baoxiang Gao, Zhixing Chen, Meiqi Li, Peng Xi

## Main text

(The Innovation *6*, 100757; February 3, 2025)

The originally published version of this paper regrettably contained some typos. First, “structure illumination microscopy” should have been written as “structured illumination microscopy” throughout the text, including in the article title, graphical abstract, the summary, and the main text. Second, in Figure 1A, “iFFT” should be written as “FFT.” Third, in Video S2, the labels “FO” and “Open” were placed incorrectly; FO is the high-quality reconstruction result, while Open contains reconstruction artifact.

The authors apologize for these errors, and these have been corrected in the paper online.

