# Supplementary material for: Fast reconstruction and optical-sectioning three-dimensional structured illumination microscopy
Source: Innovation (Camb). 2025 Jan 12;6(2):100757. doi: 10.1016/j.xinn.2024.100757 (PMC11846033; doi:10.1016/j.xinn.2024.100757)
Supplement: Document S1. Figures S1–S6, Table S1, and Notes S1–S3 [file mmc1.pdf]

The Innovation, Volume *6*

Supplemental Information

Fast reconstruction and optical-sectioning three-dimensional structured illumination microscopy

Ruijie Cao, Yaning Li, Wenyi Wang, Yunzhe Fu, Xiaoyu Bu, Dilizhatai Saimi, Jing Sun, Xichuan Ge, Shan Jiang, Yuru Pei, Baoxiang Gao, Zhixing Chen, Meiqi Li, and Peng Xi

Supplementary material of "Fast-reconstruction and optical-sectioning three-dimensional structured illumination microscopy"

Ruijie Cao[1,2], Yaning Li[1,2,3], Wenyi Wang[1,2,4], Yunzhe Fu[1,2], Xiaoyu Bu[5], Dilizhatai Saimi[6], Jing Sun[5], Xichuan Ge[4], Shan Jiang[7], Yuru Pei[8], Baoxiang Gao[4], Zhixing Chen[6,9], Meiqi Li[10,*], Peng Xi[1,2,*]

[1]Department of Biomedical Engineering, College of Future Technology, Peking University, Beijing 100871, China.

[2]National Biomedical Imaging Center, College of Future Technology, Peking University, Beijing 100871, China.

[3]China Academy of Space Technology, Beijing Institute of Space Mechanics and Electricity, 100094.

[4]Airy Technologies Co., Ltd., Beijing 100081, China.

[5]Key Laboratory of Analytical Science and Technology of Hebei Province, College of Chemistry and Materials Science, Hebei University, Baoding 071002, China.

[6]College of Future Technology, Institute of Molecular Medicine, National Biomedical Imaging Center, Beijing Key Laboratory of Cardiometabolic Molecular Medicine, Peking University, Beijing 100871, China.

[7]Institute of Biomedical Engineering, Beijing Institute of Collaborative Innovation, Beijing, China.

[8]Key Laboratory of Machine Perception (MOE), Department of Machine Intelligence, Peking University, Beijing 100871, China.

[9]Peking-Tsinghua Center for Life Science, Academy for Advanced Interdisciplinary Studies, Peking University, Beijing 100871, China.

[10]School of Life Sciences, Peking University, Beijing 100871, China.

*Correspondence: limeiqi@pku.edu.cn (M.L.), xipeng@pku.edu.cn (P.X.)

Table of Contents

Note S1. Reconstruction of traditional 3DSIM 2
Note S2. Principle of FO-3DSIM 4
Note S3. The relationship between traditional 3DSIM and FO-3DSIM 7
Supplementary Figures 10
Supplementary Movies 16
Supplementary Tables 18

Note S1. Reconstruction of traditional 3DSIM

Traditional 3DSIM reconstructions are based on the Wiener-3DSIM flowchart. The illumination distribution $I_{\theta,\varphi}(r,z)$ of 3DSIM in phase φ and angle θ can expressed as the sum of zero, first, and second harmonics of cosine functions with certain weight:

$$I_{\theta,\varphi}(r,z) = I_0 \cdot [1 + a_1(z) \cdot \cos(\omega r + \varphi) + a_2 \cdot \cos(2\omega r + 2\varphi)] \tag{1-1}$$

Where I_0 is the illumination intensity, $a_1(z) = a_1 \cos(2\pi\omega_z z)$, a_1 and a_2 are the modulation depths of the first and second harmonics, ω and ω_z are the spatial frequency in the *xoy* and *xoz* plane, (r,z) is the position parameter. Then, the emission distribution $D_{\theta,\varphi}(r,z)$ of sample $S(r,z)$ can be expressed as:

$$D_{\theta,\varphi}(r,z) = \left[S(r,z) \cdot I_{\theta,\varphi}(r,z)\right] * H(r,z) \tag{1-2}$$

Where $S(r,z)$ is the sample distribution, $H(r,z)$ is the point spread function, $*$ is the convolution function. Further, its 3D frequency domain expression can be given by:

$$\begin{aligned} D_{\theta,\varphi}(k_r,k_z) &= \left[S(k_r,k_z) \otimes I_{\theta,\varphi}(k_r,k_z)\right] \cdot H(r,z) \\ &= I_0 \begin{Bmatrix} O(k_r,k_z) + a_1(k_z) \cdot \left[S(k_r \pm \omega, k_z - \omega_z)e^{j\varphi} + S(k_r \pm \omega, k_z + \omega_z)e^{-j\varphi})e^{-j\varphi}\right] \\ +a_2 \cdot \left[S(k_{x,y} - 2\omega, k_z)e^{j2\varphi} + S(k_{x,y} + 2\omega, k_z)e^{-j2\varphi}\right] \end{Bmatrix} \cdot H(k_{x,y},k_z) \end{aligned} \tag{1-3}$$

In each angle of the illumination pattern, five even phases should be used to resolve the five components $S(k_r,k_z)$, $S(k_r \pm \omega, k_z - \omega_z) + S(k_r \pm \omega, k_z + p_z)$ and $S(k_r \pm 2\omega, \omega_z)$. Therefore, five equations can be listed in the format of the matrix:

$$\begin{bmatrix} D_{\theta,\varphi_1}(k) \\ D_{\theta,\varphi_2}(k) \\ D_{\theta,\varphi_3}(k) \\ D_{\theta,\varphi_4}(k) \\ D_{\theta,\varphi_5}(k) \end{bmatrix} = I_0 A \begin{bmatrix} S(k_r,k_z) \cdot H(k_{x,y},k_z) \\ [S(k_r - \omega, k_z - p_z) + S(k_r - \omega, k_z + p_z)] \cdot H(k_r,k_z) \\ [S(k_r + \omega, k_z - p_z) + S(k_r + \omega, k_z + p_z)] \cdot H(k_r,k_z) \\ S(k_r - 2\omega, k_z) \cdot H(k_r,k_z) \\ S(k_r + 2\omega, k_z) \cdot H(k_r,k_z) \end{bmatrix} \tag{1-4}$$

Where $A = \begin{bmatrix} a_0 & a_1 e^{j\varphi_1} & a_1 e^{-j\varphi_1} & a_2 e^{j2\varphi_1} & a_2 e^{-j2\varphi_1} \\ a_0 & a_1 e^{j\varphi_2} & a_1 e^{-j\varphi_2} & a_2 e^{j2\varphi_2} & a_2 e^{-j2\varphi_2} \\ a_0 & a_1 e^{j\varphi_3} & a_1 e^{-j\varphi_3} & a_2 e^{j2\varphi_3} & a_2 e^{-j2\varphi_3} \\ a_0 & a_1 e^{j\varphi_4} & a_1 e^{-j\varphi_4} & a_2 e^{j2\varphi_4} & a_2 e^{-j2\varphi_4} \\ a_0 & a_1 e^{j\varphi_5} & a_1 e^{-j\varphi_5} & a_2 e^{j2\varphi_5} & a_2 e^{-j2\varphi_5} \end{bmatrix}$, and the five frequency components $C_{0,\pm1,\pm2}(k_r,k_z)$ can be expressed as:

$$\begin{bmatrix} C_0(k_r,k_z) \\ C_{-1}(k_r,k_z) \\ C_{+1}(k_r,k_z) \\ C_{-2}(k_r,k_z) \\ C_{+2}(k_r,k_z) \end{bmatrix} = \begin{bmatrix} S(k_r,k_z) \cdot H(k_{x,y},k_z) \\ \left[S(k_r - p_{x,y}, k_z - p_z) + S(k_r - p_{x,y}, k_z + p_z)\right] \cdot H(k_r,k_z) \\ \left[S(k_r + p_{x,y}, k_z - p_z) + S(k_r + p_{x,y}, k_z + p_z)\right] \cdot H(k_r,k_z) \\ S(k_r - 2p_{x,y}, k_z) \cdot H(k_r,k_z) \\ S(k_r + 2p_{x,y}, k_z) \cdot H(k_r,k_z) \end{bmatrix} = \frac{1}{I_0} A^{-1} \begin{bmatrix} D_{\theta,\varphi_0}(k_r,k_z) \\ D_{\theta,\varphi_1}(k_r,k_z) \\ D_{\theta,\varphi_2}(k_r,k_z) \\ D_{\theta,\varphi_3}(k_r,k_z) \\ D_{\theta,\varphi_4}(k_r,k_z) \end{bmatrix} \tag{1-5}$$

Where $[\cdot]^{-1}$ is the symbol of the matrix inverse transform. Then five frequency components $\boldsymbol{C}_n(k_r,k_z)$ are

shifted to the correct position $C_{ns}(k_r, k_z)$ laterally. $n = 0,\ \pm1, \pm2$ represents the index of five frequency components.

$$C_{ns}(k_r, k_z) = C_n(k_r, k_z)\ , n = 0$$
$$C_{ns}(k_r, k_z) = F\{F^{-1}[C_n(k_r, k_z)] \cdot e^{-jn2\pi p_{x,y}}\}\ , n = -1, -2$$
$$C_{ns}(k_r, k_z) = F\{F^{-1}[C_n(k_r, k_z)] \cdot e^{jn2\pi p_{x,y}}\}\ , n = +1, +2$$

(1-6)

Then, the super-resolution image can be expressed as:

$$C_{SR}(k_r, k_z) = \sum_{n=-2}^{2} C_{ns}(k_r, k_z) \quad (1\text{-}7)$$

The detailed spectrum optimization including notch-filter, Weiner filter, or two-step filter is dismissed here, as seen in previous work. In the process of traditional reconstruction. The most time-consuming steps are the frequency separation and components shift, where the 3D matrix is involved to conduct the Fourier and inverse Fourier transform. Moreover, the 3D Fourier transform and its optimization steps usually need more than 6 layers in the step of frequency shift to guarantee correctness. Fewer layers to conduct frequency operation may change the final results in the spatial domain, making it difficult to reconstruct fewer *z*-layers.

Note S2. Principle of FO-3DSIM

Traditional 3DSIM reconstruction is based on frequency separation. Here, we develop FO-3DSIM based on spatial reconstruction. According to equation (1-2), when the illumination pattern has a phase shift r_i in *xoy* plane, the emission distribution $D_{\theta,\varphi}(r,z)$ can be further expressed as:

$$D_{\theta,\varphi}(r,z) \ = S(r,z)\cdot I_\theta(r'-r_i,z) * H(r,z) \tag{2-1}$$

Where r' is the corrected position parameter. Due to the process of SIM being linear, we can assume there exist coefficients $o_{\theta,i}(r,z)$, *i* = 1, 2, 3, 4, 5, that makes the following equation always true for any $O(r,z)$ and $H(r,z)$:

$$SR_{0,\theta}(r,z) = \sum_{i=1}^{5} o_{\theta,i}(r,z)\cdot D_{\theta,\varphi}(r,z) \tag{2-2}$$

Where $SR_{0,\theta}(r,z)$ denotes the initial super-resolution image in angle θ. And effective PSF can be obtained by multiplying the wide-filed PSF $H(r,z)$ with a modulation function $1+\cos(\omega_z z)\cdot\cos(\omega r)+\cos(2\omega r)$. So, $SR_{0,\theta}(r,z)$ can also written as:

$$SR_{0,\theta}(r,z) = S(r,z) * \{[1+\cos(\omega_z z)\cdot\cos(\omega r)+\cos(2\omega r)]\cdot H(r,z)\} \tag{2-3}$$

Let equation (2-2) equals to equation (2-3). And the illumination pattern can be expressed as: $I_\theta(r'-r_i,z) = I_0\cdot[1+a_1\cdot\cos(\omega_z z)\cdot\cos(\omega r'+\varphi-\omega r_i)+a_2\cdot\cos\,(2\omega r'+2\varphi-2\omega r_i)]$. We can get the following equation:

$$\begin{aligned} &I_0\sum_{i=1}^{5} e_{\theta,i}(r,z)\cdot[1+a_1\cdot\cos(\omega_z z)\cdot\cos(\omega r'+\varphi-\omega r_i)+a_2\cdot\cos(2\omega r'+2\varphi-2\omega r_i)] \\ &= 1+\cos(\omega_z z)\cdot\cos(\omega r-\omega r')+\cos(2\omega r-2\omega r') \\ &= 1+\cos(\omega_z z)\cdot\cos[(\omega r+\varphi)-(\omega r'+\varphi)]+\cos[2(\omega r+\varphi)-2(\omega r'+\varphi)] \end{aligned} \tag{2-4}$$

We rewrite both side of equation (2-4) as functions of $\cos(\omega r'+\varphi)$ and $\sin(\omega r'+\varphi)$, equation (2-4) can be written as:

$$\begin{aligned} &I_0\sum_{i=1}^{5} o_{\theta,i}(r,z)+a_1\cos(\omega_z z)\cdot I_0\sum_{i=1}^{5} o_{\theta,i}(r,z)\cdot[\cos(\omega r'+\varphi)\cos(\omega r_i)+\sin(\omega r'+\varphi)\cdot\sin(\omega r_i)]+ \\ &a_2 I_0\sum_{i=1}^{5} o_{\theta,i}(r,z)\cdot[\cos(2\omega r'+2\varphi)\cdot\cos(2\omega r_i)+\sin(2\omega r'+2\varphi)\cdot\sin(2\omega r_i)] \\ &= 1+\cos(\omega_z z)\cdot[\cos(\omega r+\varphi)\cdot\cos(\omega r'+\varphi)+\sin(\omega r+\varphi)\cdot\sin(\omega r'+\varphi)] \\ &+\cos(2\omega r+2\varphi)\cdot\cos(2\omega r'+2\varphi)+\sin(2\omega r+2\varphi)\cdot\sin(2\omega r'+2\varphi) \end{aligned} \tag{2-5}$$

Considering the orthogonality of $\cos(\omega r'+\varphi)$ and $\sin(\omega r'+\varphi)$, we can get the following equations:

$$I_0\sum_{i=1}^{5} o_{\theta,1}(r,z) = 1$$

$$a_1 I_0\sum_{i=1}^{5} o_{\theta,2}(r,z)\cdot\cos\,(\omega r_i) = \cos(\omega r+\varphi)$$

$$a_1 I_0\sum_{i=1}^{5} o_{\theta,3}(r,z)\cdot\sin\,(\omega r_i) = \sin(\omega r+\varphi)$$

$$a_2 I_0 \sum_{i=1}^{5} o_{\theta,4}(r,z) \cdot \cos(2\omega r_i) = \cos(2\omega r + 2\varphi)$$

$$a_2 I_0 \sum_{i=1}^{5} o_{\theta,5}(r,z) \cdot \sin(2\omega r_i) = \sin(2\omega r + 2\varphi) \tag{2-6}$$

To resolve the coefficients $c_{\theta,i}(r,z)$, we assume the phase shift ωr_i is $0, \frac{2}{5}\pi, \frac{4}{5}\pi, \frac{6}{5}\pi, \frac{8}{5}\pi$. And the following linear equation should be resolved:

$$\begin{bmatrix} o_{\theta,1}(r,z) \\ o_{\theta,2}(r,z) \\ o_{\theta,3}(r,z) \\ o_{\theta,4}(r,z) \\ o_{\theta,5}(r,z) \end{bmatrix} \begin{bmatrix} 1 & 1 & 1 & 1 & 1 \\ \cos 0 & \cos\frac{2}{5}\pi & \cos\frac{4}{5}\pi & \cos\frac{6}{5}\pi & \cos\frac{8}{5}\pi \\ \sin 0 & \sin\frac{2}{5}\pi & \sin\frac{4}{5}\pi & \sin\frac{6}{5}\pi & \sin\frac{8}{5}\pi \\ \cos 0 & \cos\frac{4}{5}\pi & \cos\frac{8}{5}\pi & \cos\frac{12}{5}\pi & \cos\frac{16}{5}\pi \\ \sin 0 & \sin\frac{4}{5}\pi & \sin\frac{8}{5}\pi & \sin\frac{12}{5}\pi & \sin\frac{16}{5}\pi \end{bmatrix} = \begin{bmatrix} \frac{1}{I_0} \\ \frac{\cos(\omega r+\varphi)}{a_1 I_0} \\ \frac{\sin(\omega r+\varphi)}{a_1 I_0} \\ \frac{\cos(2\omega r+2\varphi)}{a_2 I_0} \\ \frac{\sin(2\omega r+2\varphi)}{a_2 I_0} \end{bmatrix} \tag{2-7}$$

Because 3DSIM is suitable for thick sample imaging, we subtract the wide-field image in this step and add the optical-sectioning results in the following steps. Equation (2-7) can be rewritten as:

$$\begin{bmatrix} o_{\theta,1}(r,z) \\ o_{\theta,2}(r,z) \\ o_{\theta,3}(r,z) \\ o_{\theta,4}(r,z) \\ o_{\theta,5}(r,z) \end{bmatrix} \begin{bmatrix} 0 & 0 & 0 & 0 & 0 \\ \cos 0 & \cos\frac{2}{5}\pi & \cos\frac{4}{5}\pi & \cos\frac{6}{5}\pi & \cos\frac{8}{5}\pi \\ \sin 0 & \sin\frac{2}{5}\pi & \sin\frac{4}{5}\pi & \sin\frac{6}{5}\pi & \sin\frac{8}{5}\pi \\ \cos 0 & \cos\frac{4}{5}\pi & \cos\frac{8}{5}\pi & \cos\frac{12}{5}\pi & \cos\frac{16}{5}\pi \\ \sin 0 & \sin\frac{4}{5}\pi & \sin\frac{8}{5}\pi & \sin\frac{12}{5}\pi & \sin\frac{16}{5}\pi \end{bmatrix} = \begin{bmatrix} \frac{1}{I_0} \\ \frac{\cos(\omega r+\varphi)}{a_1 I_0} \\ \frac{\sin(\omega r+\varphi)}{a_1 I_0} \\ \frac{\cos(2\omega r+2\varphi)}{a_2 I_0} \\ \frac{\sin(2\omega r+2\varphi)}{a_2 I_0} \end{bmatrix} \tag{2-8}$$

It can be seen that coefficients $o_{\theta,i}(r,z)$ have no relevance of *z*, so they only depend on the lateral parameter *r*. So, the super-resolution image $SR_{0,-}(r,z)$ (minus the 0th component) can be expressed as:

$$SR_{0,-}(r,z) = \sum_{\theta=1}^{3} \sum_{i=1}^{5} o_{\theta,i}(r,z) \cdot D_{\theta,i}(r,z) \tag{2-9}$$

Then, to reduce the honeycomb artifacts, we use a notch filter to suppress the high-frequency peaks. The notch filter $notch(x,y,z,n)$ is the same form as Open-3DSIM, but it did not notch the center frequency component because the minus of wide-field image. The notched frequency domain of super-resolution image can be expressed as:

$$F_{SR_{0,-}}(r,z) = \left[\prod_{n=-2,-1,1,2} notch(x,y,z,n)\right] \cdot F[SR_{0,-}(r,z)] \tag{2-10}$$

Where $notch(x,y,z,0) = 1 - d \cdot exp\left[\left(\frac{x^2+y^2}{|p_{x,y}|^2} + \frac{z^2}{|p_z|^2}\right)/2/w^2\right]$ is the notch-filter designed according to the estimated frequency vector on the *xoy* and *yoz* plane, $notch(x,y,z,n)$ is the corresponding shifted notch filter in frequency-domain position on the base of $notch(x,y,z,0)$, d and w are the notch depth and width, respectively, $F[\cdot]$ is the symbol of Fourier transform, $\prod[\cdot]$ is the multiplicative multiplication symbol.

Then, we use a two-step spectrum filter of $Filter1(k) = \frac{Apo}{OTF_{notch}+w_1^2}$ and $Filter2(k) = \frac{Apo}{OTF_{notch}+w_2^2}$ similar to Open-3DSIM to minimize the artifacts of reconstruction, where $OTF_{notch} = \sum_{n=-2,-1,1,2} m(n) \cdot OTF(x,y,z,n) \cdot$

$notch(x,y,z,n)$, $m(n)$ is the weight coefficient of different Fourier orders, $OTF(x,y,z,n)$ is the leaky-cone-shaped optical transfer function (OTF) shifted to the n-th place in the frequency domain, w_1 and w_2 are the parameters to adjust the filters. The filtered super-resolution image $SR_1(r,z)$ can be expressed as:

$$SR_1(r,z) = F^{-1}\left[Filter1(k) \cdot Filter2(k) \cdot F_{SR_{0,-}}(r,z)\right] \quad (2\text{-}11)$$

Previously, we subtracted the wide-filed image to suppress the defocused background. Here, we use the HiLo algorithm to remove the defocused background. HiLo microscopy uses one image with low spectrum spread patterned illumination and one wide-field image to get a background removed image. 3DSIM has lower harmonics (~1.5 spread spectrum) compared with 2DSIM, so introducing HiLo into 3DSIM corresponds to the requirement of HiLo. Assuming the raw patterned image with angle θ and i-th phase is $D_{\theta,i}(r,z)$. The HiLo image $OS(r,z)$ can be expressed as:

$$OS(r,z) = \sum_{\theta=1}^{3}\left\{Lo\left[D_{\theta,1}(r,z)\right] + Hi\left[\sum_{i=1}^{5} D_{\theta,i}(r,z)/5\right]\right\}/3 \quad (2\text{-}12)$$

Where $Lo[\cdot]$ and $Hi[\cdot]$ are the low-pass and high-pass filter, respectively, and we average the three angles of illumination to avoid light intensity difference in three angles. At last, the final super-resolution image $SR_2(r,z)$ can be expressed as:

$$SR_2(r,z) = SR_1(r,z) + OS(r,z) \quad (2\text{-}13)$$

Note S3. The relationship between traditional 3DSIM and FO-3DSIM

Here, we will discuss that the principle of FO-3DSIM is identical to traditional 3DSIM. The spatial reconstruction will not sacrifice the fidelity and resolution of traditional 3DSIM. We first rewrite equation (2-7) into the following equation:

$$\begin{bmatrix} o_1(r,z) \\ o_2(r,z) \\ o_3(r,z) \\ o_4(r,z) \\ o_5(r,z) \end{bmatrix} = \begin{bmatrix} O_{11} & O_{12} & O_{13} & O_{14} & O_{15} \\ O_{21} & O_{22} & O_{23} & O_{24} & O_{25} \\ O_{31} & O_{32} & O_{33} & O_{34} & O_{35} \\ O_{41} & O_{42} & O_{43} & O_{44} & O_{45} \\ O_{51} & O_{52} & O_{53} & O_{54} & O_{55} \end{bmatrix} \begin{bmatrix} \frac{1}{I_0} \\ \frac{\cos(\omega r+\varphi)}{a_1 I_0} \\ \frac{\sin(\omega r+\varphi)}{a_1 I_0} \\ \frac{\cos(2\omega r+2\varphi)}{a_2 I_0} \\ \frac{\sin(2\omega r+2\varphi)}{a_2 I_0} \end{bmatrix} \tag{3-1}$$

Where $\begin{bmatrix} O_{11} & O_{12} & O_{13} & O_{14} & O_{15} \\ O_{21} & O_{22} & O_{23} & O_{24} & O_{25} \\ O_{31} & O_{32} & O_{33} & O_{34} & O_{35} \\ O_{41} & O_{42} & O_{43} & O_{44} & O_{45} \\ O_{51} & O_{52} & O_{53} & O_{54} & O_{55} \end{bmatrix} = \begin{bmatrix} 1 & 1 & 1 & 1 & 1 \\ \cos 0 & \cos\frac{2}{5}\pi & \cos\frac{4}{5}\pi & \cos\frac{6}{5}\pi & \cos\frac{8}{5}\pi \\ \sin 0 & \sin\frac{2}{5}\pi & \sin\frac{4}{5}\pi & \sin\frac{6}{5}\pi & \sin\frac{8}{5}\pi \\ \cos 0 & \cos\frac{4}{5}\pi & \cos\frac{8}{5}\pi & \cos\frac{12}{5}\pi & \cos\frac{16}{5}\pi \\ \sin 0 & \sin\frac{4}{5}\pi & \sin\frac{8}{5}\pi & \sin\frac{12}{5}\pi & \sin\frac{16}{5}\pi \end{bmatrix}^{-1}$ denotes the intermediate matrix.

According to the equation (2-10), the super-resolution image $SR_0(r,z)$ can be further expressed as:

$$\begin{aligned} SR_0(r,z) &= \textstyle\sum_{i=1}^{5} o_i(r,z)\cdot D_i(r,z) \\ &= \tfrac{1}{I_0}\textstyle\sum_{i=1}^{5} O_{i1}D_i(r,z) + \tfrac{1}{a_1 I_0}\sum_{i=1}^{5} O_{i2}D_i(r,z)\cdot\cos(\omega r+\varphi) + \tfrac{1}{a_1 I_0}\sum_{i=1}^{5} O_{i3}D_i(r,z)\cdot\sin(\omega r+\varphi) \\ &\quad + \tfrac{1}{a_2 I_0}\textstyle\sum_{i=1}^{5} O_{i4}D_i(r,z)\cdot\cos(2\omega r+2\varphi) + \tfrac{1}{a_2 I_0}\sum_{i=1}^{5} O_{i5}D_i(r,z)\cdot\sin(2\omega r+2\varphi) \end{aligned} \tag{3-2}$$

To simplify the equation above, we further define $E_i(r,z)$ as:

$$\begin{aligned} E_0(r,z) &= \tfrac{1}{I_0}\textstyle\sum_{i=1}^{5} O_{i1}D_i(r,z) \\ E_1(r,z) &= \tfrac{1}{a_1 I_0}\textstyle\sum_{i=1}^{5} O_{i2}D_i(r,z) \\ E_2(r,z) &= \tfrac{1}{a_1 I_0}\textstyle\sum_{i=1}^{5} O_{i3}D_i(r,z) \\ E_3(r,z) &= \tfrac{1}{a_2 I_0}\textstyle\sum_{i=1}^{5} O_{i4}D_i(r,z) \\ E_4(r,z) &= \tfrac{1}{a_2 I_0}\textstyle\sum_{i=1}^{5} O_{i5}D_i(r,z) \end{aligned} \tag{3-3}$$

Without loss of generality, let $I_0 = 1$, the super-resolution image $SR_0(r,z)$ can be simplified as:

$$\begin{aligned} SR_0(r,z) &= E_0(r,z) + E_1(r,z)\cos(\omega r+\varphi) + E_2(r,z)\sin(\omega r+\varphi) + E_3(r,z)\cos(2\omega r+2\varphi) \\ &\quad + E_4(r,z)\sin(2\omega r+2\varphi) \end{aligned}$$

(3-4)

Then, equation (2-3) can be expressed as $D_i(r,z) = S(r,z) \cdot [1 + \cos(\omega r + \varphi_i) + \cos(2\omega r + 2\varphi_i)] * H(r,z)$, $R_i(r,z)$ can be expressed as:

$$E_0(r,z) = S(r,z) * H(r,z)$$

$$E_1(r,z) = [S(r,z) \cdot \cos(\omega r + \varphi)] * H(r,z)$$

$$E_2(r,z) = [S(r,z) \cdot \sin(\omega r + \varphi)] * H(r,z)$$

$$E_4(r,z) = [S(r,z) \cdot \cos(2\omega r + 2\varphi)] * H(r,z)$$

$$E_5(r,z) = [S(r,z) \cdot \sin(2\omega r + 2\varphi)] * H(r,z) \tag{3-5}$$

According to the equation that $\cos(\omega r + \varphi) = \frac{e^{j(\omega r+\varphi)} + e^{-j(\omega r+\varphi)}}{2}$, $E_1(r,z)$ can be additionally expressed as:

$$\begin{aligned} E_1(r,z) &= \left[S(r,z) \cdot \frac{e^{j(\omega r+\varphi)} - e^{-j(\omega r+\varphi)}}{2}\right] * H(r,z) \\ &= \frac{e^{j\varphi}}{2}\left[S(r,z)e^{j\omega r}\right] * H(r,z) + \frac{e^{-j\varphi}}{2}\left[S(r,z)e^{-j\omega r}\right] * H(r,z) \\ &= \frac{e^{j\varphi}}{2}F^{-1}[S(k-\omega,z) \cdot H(k,z)] + \frac{e^{-j\varphi}}{2}F^{-1}[S(k+\omega,z) \cdot H(k,z)] \end{aligned}$$

(3-6)

Similarly, $E_2(r,z)$, $E_3(r,z)$, $E_4(r,z)$ can be expressed as:

$$E_2(r,z) = \frac{e^{j\varphi}}{2j}F^{-1}[S(k-\omega,z) \cdot H(k,z)] - \frac{e^{-j\varphi}}{2j}F^{-1}[S(k+\omega,z) \cdot H(k,z)]$$

$$E_3(r,z) = \frac{e^{j2\varphi}}{2}F^{-1}[S(k-2\omega,z) \cdot H(k,z)] + \frac{e^{-j2\varphi}}{2}F^{-1}[S(k+2\omega,z) \cdot H(k,z)]$$

$$E_4(r,z) = \frac{e^{j2\varphi}}{2j}F^{-1}[S(k-2\omega,z) \cdot H(k,z)] - \frac{e^{-j2\varphi}}{2j}F^{-1}[S(k+2\omega,z) \cdot H(k,z)] \tag{3-7}$$

After solving the linear formula of equation (3-6) and (3-7), $S(k \pm \omega, z) \cdot H(k,z)$ and $S(k \pm 2\omega, z) \cdot H(k,z)$ can be resolved:

$$S(k-\omega,z) \cdot H(k,z) = e^{-j\varphi} \cdot F[E_1(r,z) + jE_2(r,z)]$$

$$S(k+\omega,z) \cdot H(k,z) = e^{j\varphi} \cdot F[E_1(r,z) - jE_2(r,z)]$$

$$S(k-2\omega,z) \cdot H(k,z) = e^{-j2\varphi} \cdot F[E_3(r,z) + jE_4(r,z)]$$

$$S(k+2\omega,z) \cdot H(k,z) = e^{j2\varphi} \cdot F[E_3(r,z) + jE_4(r,z)]$$

(3-8)

In traditional 3DSIM reconstruction, the frequency components $M_0(k,z)$, $M_{-1}(k,z)$ can be expressed as:

$$M_0(k,z) = S(k,z) \cdot H(k,z)$$

$$M_{-1}(k,z) = \frac{1}{2}S(k,z) \cdot H(k-\omega,z)$$

$$= \frac{1}{2} F\left\{F^{-1}[S(k+\omega, z) \cdot H(k, z)] e^{j\omega r}\right\}$$
$$= \frac{1}{2} F\left\{F^{-1}\left[e^{j\varphi} \cdot F[E_1(r, z) - jE_2(r, z)]\right] e^{j\omega r}\right\}$$
$$= \frac{1}{2} F\left\{[E_1(r, z) - jE_2(r, z)] e^{j(\omega r+\varphi)}\right\}$$

(3-9)

Similarly, $M_{+1}(k,z)$, $M_{-2}(k,z)$, and $M_{+2}(k,z)$ can be expressed as:

$$M_{+1}(k, z) = \frac{1}{2} F\left\{[E_1(r, z) + jE_2(r, z)] e^{-j(\omega r+\varphi)}\right\}$$
$$M_{-2}(k, z) = \frac{1}{2} F\left\{[E_3(r, z) - jE_4(r, z)] e^{j2(\omega r+\varphi)}\right\}$$
$$M_{+2}(k, z) = \frac{1}{2} F\left\{[E_3(r, z) + jE_4(r, z)] e^{-j2(\omega r+\varphi)}\right\}$$

(3-10)

After considering equation (3-9) and (3-10), the super-resolution image can be expressed as the following equation in traditional 3DSIM reconstruction:

$$SR_0(r, z) = F^{-1}\{M_0(k, z) + M_{-1}(k, z) + M_{+1}(k, z) + M_{-2}(k, z) + M_{+2}(k, z)\}$$
$$= F^{-1}\left\{\begin{matrix} S(k, z) \cdot H(k, z) + \frac{1}{2} F\left\{[E_1(r, z) - jE_2(r, z)] e^{j(\omega r+\varphi)}\right\} + \frac{1}{2} F\left\{[E_1(r, z) + jE_2(r, z)] e^{-j(\omega r+\varphi)}\right\} \\ + \frac{1}{2} F\left\{[E_3(r, z) - jE_4(r, z)] e^{j2(\omega r+\varphi)}\right\} + \frac{1}{2} F\left\{[E_3(r, z) + jE_4(r, z)] e^{-j2(\omega r+\varphi)}\right\} \end{matrix}\right\}$$
$$= E_0(r, z) + E_1(r, z)\cos(\omega r + \varphi) + E_2(r, z)\sin(\omega r + \varphi) + E_3(r, z)\cos(2\omega r + 2\varphi) +$$
$$E_4(r, z)\sin(2\omega r + 2\varphi)$$

(3-11)

We can find that equation (3-11) is the same formulation as equation (3-4). As a result, FO-3DSIM is equal to traditional 3DSIM mathematically.

Supplementary Figures

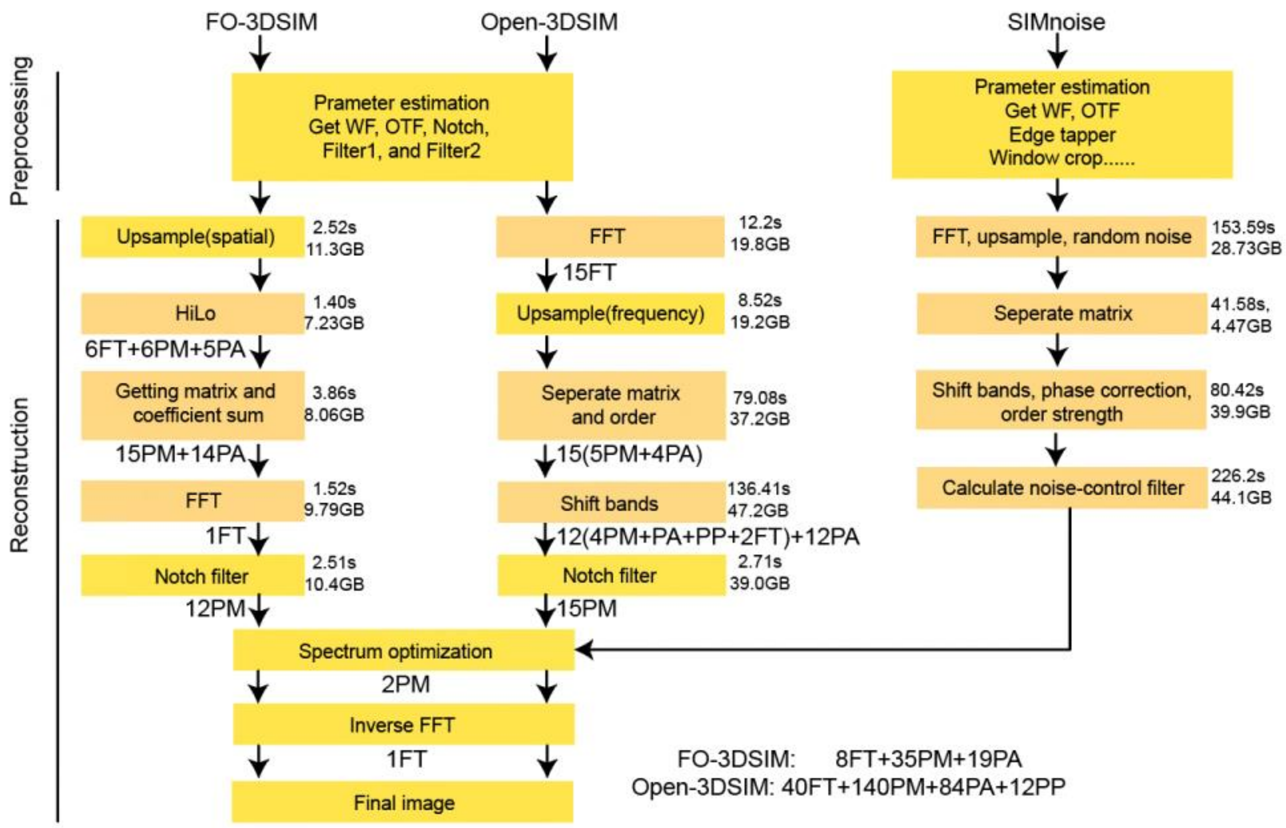

Figure S1. Comparison of reconstruction time of immediate steps in FO-3DSIM, Open-3DSIM, and SIMnoise. The image size is 512×512×41, Both FO-3DSIM, Open-3DSIM, and SIMnoise are written by CPU only for fair comparison. FT, (inverse) Fourier transforms, PM, pointwise multiplications, PA, pointwise multiplications additions, PP, pointwise power.

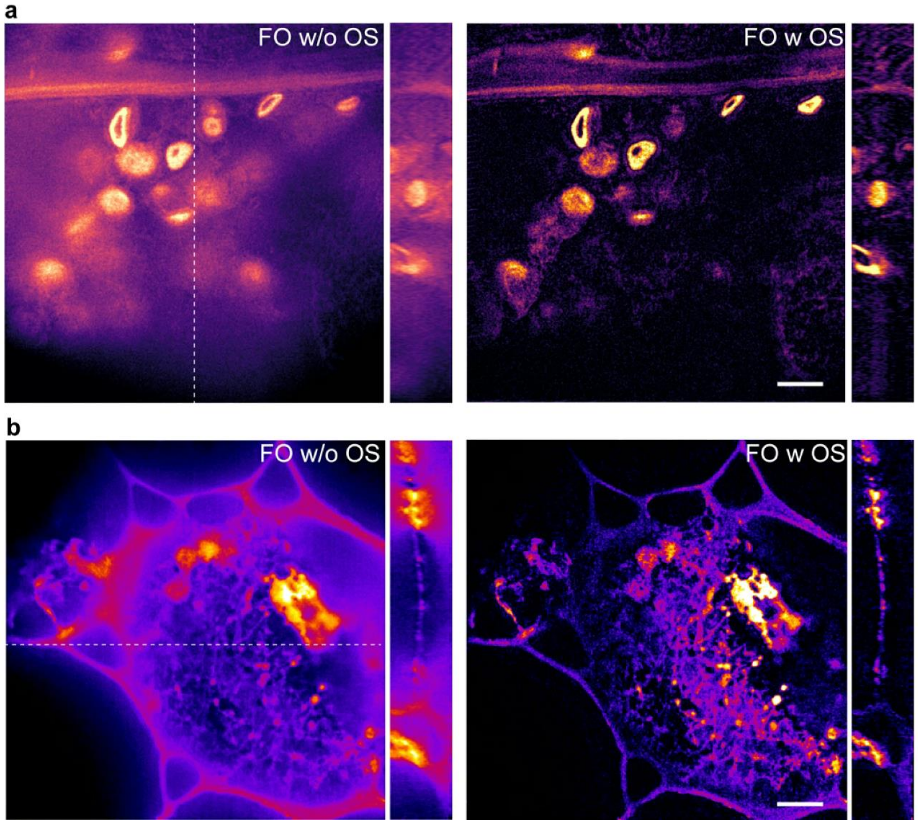

Figure S2. Effect of combining OS-SIM into FO-3DSIM. Comparison between FO-3DSIM with OS-SIM (FO w OS) and FO-3DSIM without OS-SIM (FO w/o OS). Scale bar: 4 µm.

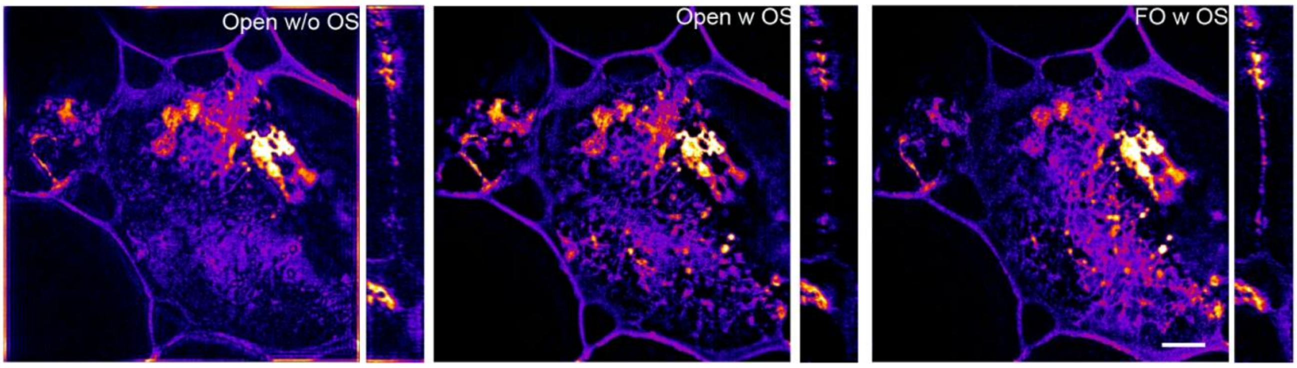

Figure S3. Traditional 3DSIM can also benefit from combining OS-SIM and 3DSIM. Scale bar: 4 μm. Axial scale: 44 layers, 125nm per layer.

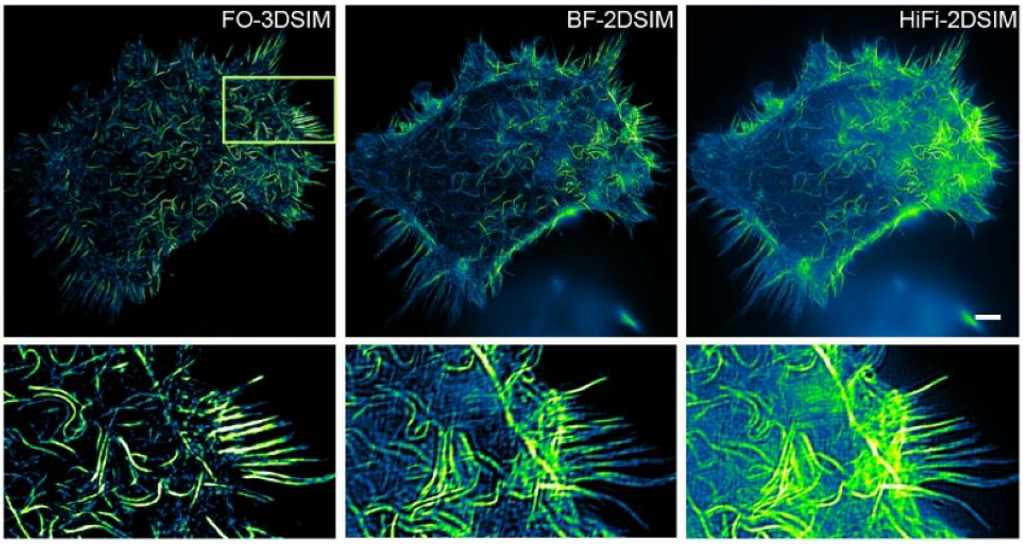

Figure S4. Comparison between FO-3DSIM and state-of-art 2DSIM results including BF-SIM and HiFi-SIM.

The data is from Fig.5(a) in the manuscript of live actin filament. Scale bar: 4 μm.

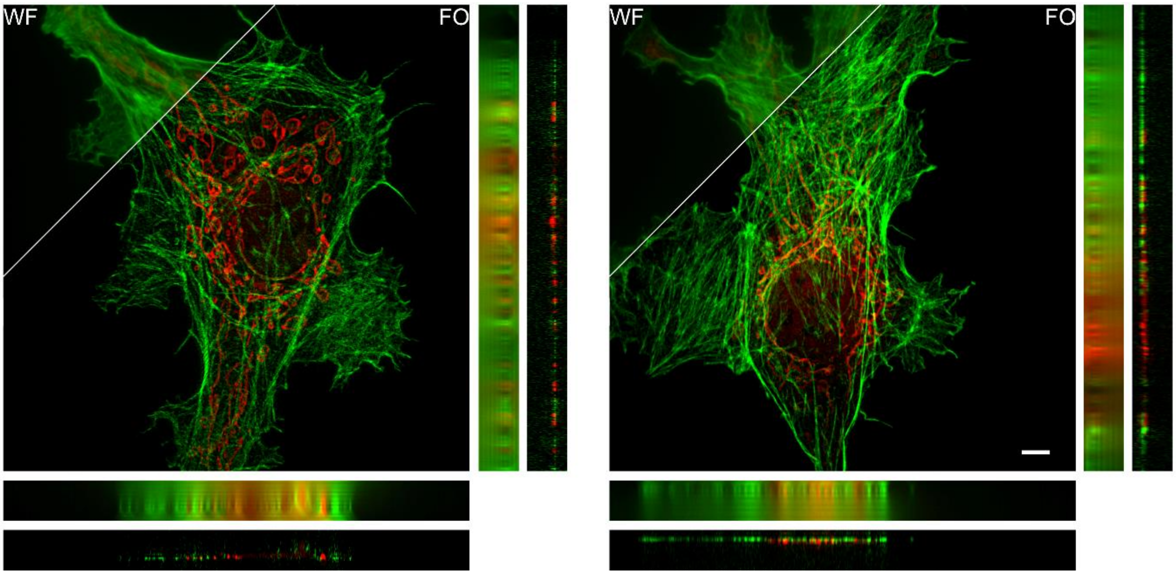

Figure S5. FO-3DSIM assists in multi-color imaging of actin filament and mitochondria. Scale bar: 4 μm.

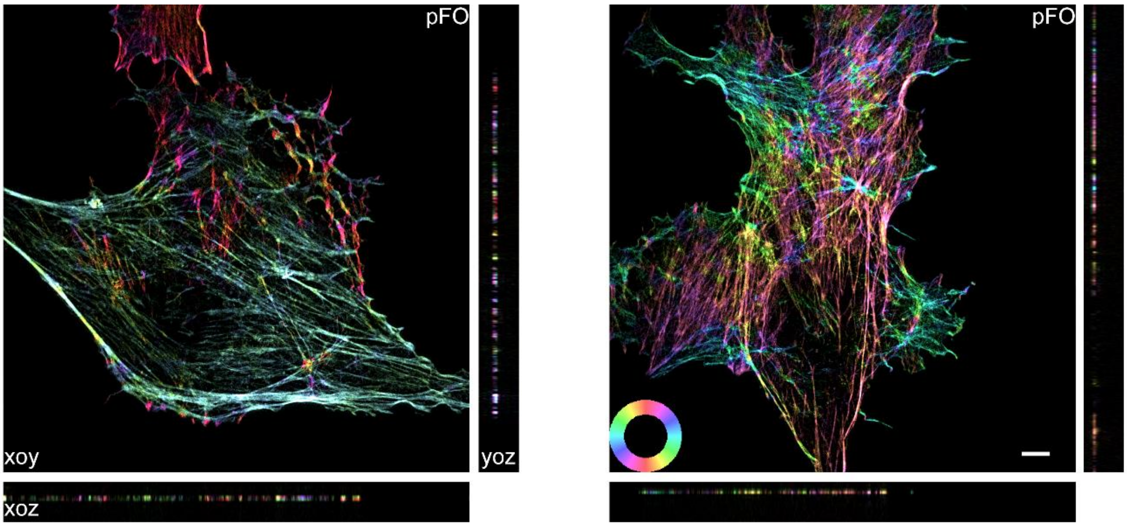

Figure S6. FO-3DSIM resolves the dipole orientation of actin filaments. Scale bar: 4 μm.

Supplementary Movies

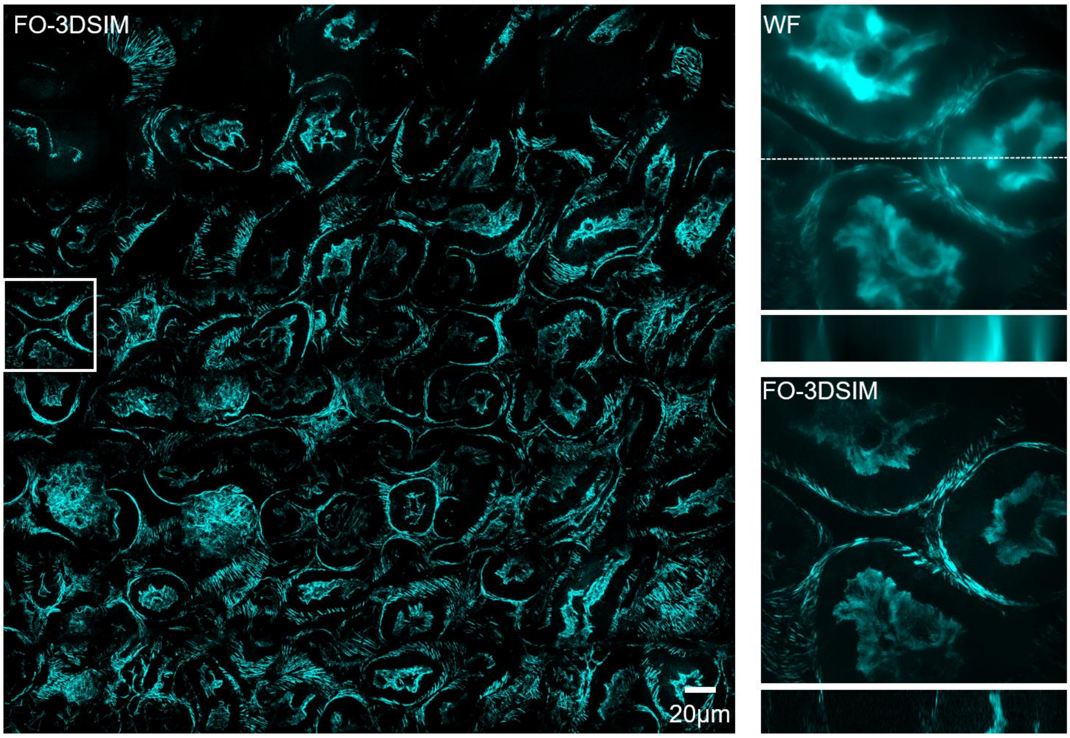

Movie S1. 3D Large-FOV super-resolution imaging.

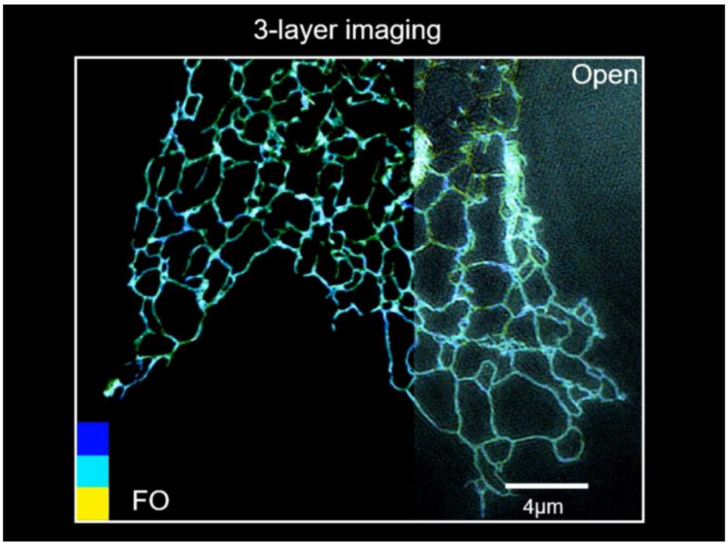

Movie S2. FO-3DSIM enables limited-layer reconstruction, and produces lower photodamage.

Supplementary Tables

Supplementary Table 1. Information of samples in this article

Figure	System	Sample	Excitation wavelength	Pixel size	NA
Fig. 1(a)	DMD-3DSIM	Black algal leaves	561nm	65nm	1.49
Fig. 2(e, f)	N-SIM2	Mouse kidney section	561nm	65nm	1.49
Fig. 3(a)	OMX	Actin	Open-source data from Open-3DSIM		
Fig. 3(c, d)	DMD-3DSIM	Black algal leaves	Open-source data from DMD-3DSIM		
Fig. 3(f)	DMD-3DSIM	Veins in oleander leaves	561nm	65nm	1.49
Fig. 3(g)	DMD-3DSIM	Mouse kidney section	561nm	65nm	1.49
Fig. 4	N-SIM2	Mouse kidney section	561nm	65nm	1.49
Fig. 5	OMX	Actin	561nm	80nm	1.40
Fig. 6	OMX	ER tubes	561nm	80nm	1.40
Fig. S2(a)	DMD-3DSIM	Black algal leaves	Open-source data from DMD-3DSIM		
Fig. S2(b)	DMD-3DSIM	Veins in oleander leaves	561nm	65nm	1.49
Fig. S3	DMD-3DSIM	Veins in oleander leaves	561nm	65nm	1.49
Fig. S4	OMX	Actin	561nm	80nm	1.40
Fig. S5	Polar-SIM	Actin + Mitochondria	561nm / 640nm	65nm	1.49
Fig. S6	Polar-SIM	Actin	561nm	65nm	1.49
