# Supplementary material for: Fast reconstruction and optical-sectioning three-dimensional structured illumination microscopy
Source: Innovation (Camb). 2025 Jan 12;6(2):100757. doi: 10.1016/j.xinn.2024.100757 (PMC11846033; doi:10.1016/j.xinn.2024.100757)
Supplement: Document S2. Article plus supplemental information [file mmc4.pdf]

OPEN ACCESS

www.the-innovation.org

Fast reconstruction and optical-sectioning three-dimensional structured illumination microscopy

Ruijie Cao,[1,2] Yaning Li,[1,2,3] Wenyi Wang,[1,2,4] Yunzhe Fu,[1,2] Xiaoyu Bu,[5] Dilizhatai Saimi,[6] Jing Sun,[5] Xichuan Ge,[4] Shan Jiang,[7] Yuru Pei,[8] Baoxiang Gao,[4] Zhixing Chen,[1,2,9] Meiqi Li,[10,*] and Peng Xi[1,2,*]

*Correspondence: limeiqi@pku.edu.cn (M.L.); xipeng@pku.edu.cn (P.X.)

Received: July 1, 2024; Accepted: December 9, 2024; Published Online: January 12, 2025; https://doi.org/10.1016/j.xinn.2024.100757

© 2024 The Authors. Published by Elsevier Inc. on behalf of Youth Innovation Co., Ltd. This is an open access article under the CC BY license (http://creativecommons.org/licenses/by/4.0/).

GRAPHICAL ABSTRACT

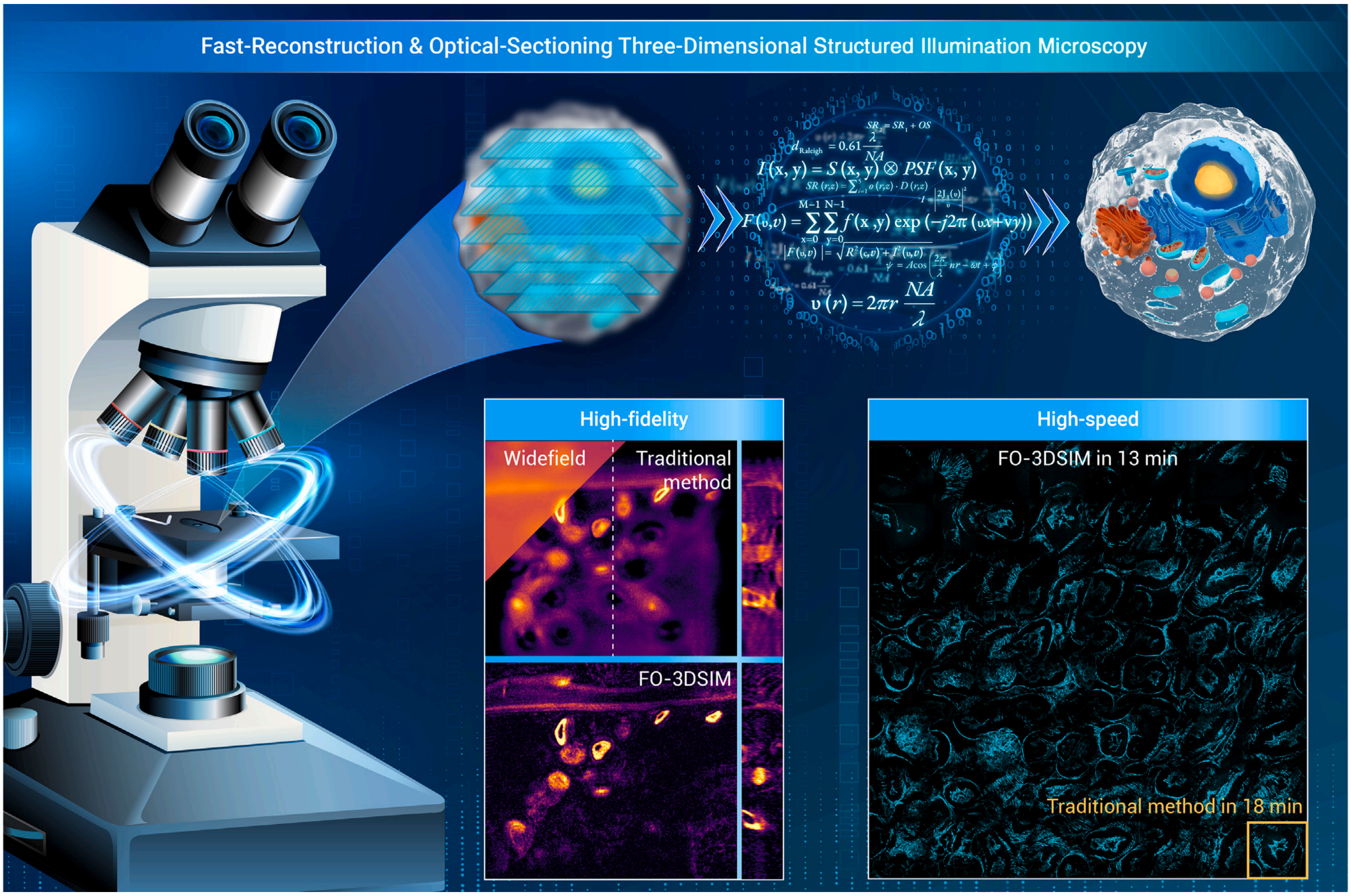

PUBLIC SUMMARY

- 3D structured illumination microscopy is enhanced integrating spatial reconstruction and optical sectioning.
- Reconstruction is speed up by nearly three orders of magnitude and fidelity has been greatly improved.
- We achieve high-throughput and near-real-time 3D super-resolution imaging with high speed and fidelity.

OPEN ACCESS

REPORT

Fast reconstruction and optical-sectioning three-dimensional structured illumination microscopy

Ruijie Cao,[1,2] Yaning Li,[1,2,3] Wenyi Wang,[1,2,4] Yunzhe Fu,[1,2] Xiaoyu Bu,[5] Dilizhatai Saimi,[6] Jing Sun,[5] Xichuan Ge,[4] Shan Jiang,[7] Yuru Pei,[8] Baoxiang Gao,[4] Zhixing Chen,[1,2,9] Meiqi Li,[10,*] and Peng Xi[1,2,*]

[1]Department of Biomedical Engineering, College of Future Technology, Peking University, Beijing 100871, China
[2]National Biomedical Imaging Center, College of Future Technology, Peking University, Beijing 100871, China
[3]China Academy of Space Technology, Beijing Institute of Space Mechanics and Electricity, Beijing 100094, China
[4]Airy Technologies Co., Ltd., Beijing 100081, China
[5]Key Laboratory of Analytical Science and Technology of Hebei Province, College of Chemistry and Materials Science, Hebei University, Baoding 071002, China
[6]College of Future Technology, Institute of Molecular Medicine, National Biomedical Imaging Center, Beijing Key Laboratory of Cardiometabolic Molecular Medicine, Peking University, Beijing 100871, China
[7]Institute of Biomedical Engineering, Beijing Institute of Collaborative Innovation, Beijing, China
[8]Key Laboratory of Machine Perception (MOE), Department of Machine Intelligence, Peking University, Beijing 100871, China
[9]Peking-Tsinghua Center for Life Science, Academy for Advanced Interdisciplinary Studies, Peking University, Beijing 100871, China
[10]School of Life Sciences, Peking University, Beijing 100871, China
*Correspondence: limeiqi@pku.edu.cn (M.L.); xipeng@pku.edu.cn (P.X.)

Received: July 1, 2024; Accepted: December 9, 2024; Published Online: January 12, 2025; https://doi.org/10.1016/j.xinn.2024.100757

© 2024 The Authors. Published by Elsevier Inc. on behalf of Youth Innovation Co., Ltd. This is an open access article under the CC BY license (http://creativecommons.org/licenses/by/4.0/).

Citation: Cao R., Li Y., Wang W., et al., (2025). Fast reconstruction and optical-sectioning three-dimensional structured illumination microscopy. The Innovation **6(2)**, 100757.

Three-dimensional structured illumination microscopy (3DSIM) is a popular method for observing subcellular/cellular structures or animal/plant tissues with gentle phototoxicity and 3D super-resolution. However, its time-consuming reconstruction process poses challenges for high-throughput imaging and real-time observation. Moreover, traditional 3DSIM typically requires more than six *z* layers for successful reconstruction and is susceptible to defocused backgrounds. This poses a great gap between single-layer 2DSIM and 6-layer 3DSIM, and limits the observation of thicker samples. To address these limitations, we developed FO-3DSIM, a novel method that integrates spatial-domain reconstruction with optical-sectioning SIM. FO-3DSIM enhances reconstruction speed by up to 855.7 times with superior performance with limited *z* layers and under high defocused backgrounds. It retains the high-fidelity, low-photon reconstruction capabilities of our previously proposed Open-3DSIM. Utilizing fast reconstruction and optical sectioning, we achieved large field-of-view (FOV) 3D super-resolution imaging of mouse kidney actin, covering a region of 0.453 mm × 0.453 mm × 2.75 μm within 23 min of acquisition and 13 min of reconstruction. Near real-time performance was demonstrated in live actin imaging with FO-3DSIM. Our approach reduces photodamage through limited *z* layer reconstruction, allowing the observation of ER tubes with just three layers. We anticipate that FO-3DSIM will pave the way for near real-time, large FOV 6D imaging, encompassing *xyz* super-resolution, multi-color, long-term, and polarization imaging with less photodamage, removed defocused backgrounds, and reduced reconstruction time.

INTRODUCTION

Recent years have witnessed the rapid development of structured illumination microscopy (SIM) due to its high spatiotemporal resolution and low optical invasiveness for live imaging.[1-5] Overall, SIM can be divided into two categories: two-dimensional structured illumination microscopy (2DSIM) and three-dimensional structured illumination microscopy (3DSIM).[3,6-8] 3DSIM can achieve double improvement in the axial plane compared with 2DSIM, making it particularly suitable for observing samples with a certain 3D structure. Although significant progress has been made in the hardware and algorithms of 2DSIM,[9-12] the development of 3DSIM technology is relatively slow due to the complex hardware system and time-consuming reconstruction process.[8]

Previously, JSFR-based SIM,[13-15] rooted in joint space and frequency reconstruction, was proposed, demonstrating a significant improvement in the reconstruction speed of 2DSIM or single-layer 3DSIM. However, multi-layer 3DSIM with double resolution in the axial plane typically demands computation times ranging from minutes to hours, exhibiting exponential growth of reconstruction time compared with 2DSIM. Current open-source 3DSIM algorithms, including our previous work Open-3DSIM, are still based on traditional frequency domain reconstruction. Due to phase separation and frequency shifts, traditional 3DSIM algorithms are both time- and memory-intensive. Furthermore, successful reconstruction with traditional 3DSIM usually requires a sufficient number of *z* layers (typically more than six),[16] which poses a great gap between single-layer 2DSIM and 6-layer 3DSIM.

Another tricky issue in 3DSIM is the defocused backgrounds. Because 3DSIM is typically used to process samples with a certain thickness, the out-of-focus background caused by factors such as scattering becomes significant.[17] To remove the out-of-focus background in 3DSIM, efforts are made from both algorithmic and hardware perspectives. For instance, Open-3DSIM suppresses low-frequency signals by applying notch filters to the center component.[16] However, this approach introduces severe artifacts if the background is overwhelmed. Selective-plane 3DSIM appears to be a better solution[18]; however, it requires special fluorescent probes and dual excitation with SIM and optical sections, which undermines the advantage of SIM's low phototoxicity.

In this work, we develop a 3DSIM reconstruction method with the advantages of fast reconstruction and optical sectioning (FO-3DSIM). FO-3DSIM reconstructs 3DSIM images in the spatial domain and optimizes results in the frequency domain by integrating 3DSIM and optical-sectioning SIM. FO-3DSIM reduces the reconstruction time by up to 855.7 times, significantly lowering computer memory requirements. Thus, it enables large field-of-view (FOV) 3D imaging and near real-time live cell observation. Moreover, it greatly suppresses the defocused background without a notch filter or additional setup, assisting thick sample imaging. Lastly, FO-3DSIM fills the gap between 2DSIM and 3DSIM by reconstructing using fewer *z* layers and is friendly to live cell imaging.

MATERIALS AND METHODS

Cell culture

COS7 cells (derived from African green monkey kidney fibroblasts and transformed with the SV40 virus gene) were cultured in high-glucose DMEM supplemented with 10% fetal bovine serum and 1% penicillin-streptomycin to prevent contamination. Cells were seeded at an appropriate density in 25 cm^2 culture flasks and incubated at 37°C in a 5% CO_2 atmosphere. Once the cells reached a confluency of 80% or higher, they were trypsinized to create a cell suspension. A suitable volume of the suspension was then seeded onto glass-bottom culture dishes. After a 12-h incubation period to allow for cell adherence. For live ER experiment, a 1 mM stock solution of ER-Tracker Green (MedChemExpress, USA) was prepared in DMSO. This stock solution was then diluted with 1 mL of DMEM culture medium to achieve a working concentration of 1 μM. Healthy cells were selected, and the existing culture medium was replaced with the prepared staining solution. The cells were incubated for 30 min at 37°C in a 5% CO_2 incubator. For live actin experiments, cells were seeded onto the coverslips (STGBD-035-1, Standard Imaging) to achieve ~70% confluence before transfection. Cytoskeletal microfilaments were labeled using the EGFP-lifeact plasmid. Transfections were executed using Lipofectamine 3000 (Invitrogen) according to the manufacturer's protocol. Cells were imaged 48 h post-transfection in a live cell workstation at 37°C and 5% CO_2.

Open-source data

The actin filament in Figure 3A is from our previously proposed Open-3DSIM. The mouse kidney section in Figure 3G is from our previously proposed DMD-3DSIM.

Open-source algorithm

In this paper, Open-3DSIM and SIMnoise are used for 3DSIM reconstruction comparisons,[16,19] and HiFi-SIM and BF-SIM are used for 2DSIM reconstruction.[11,17]

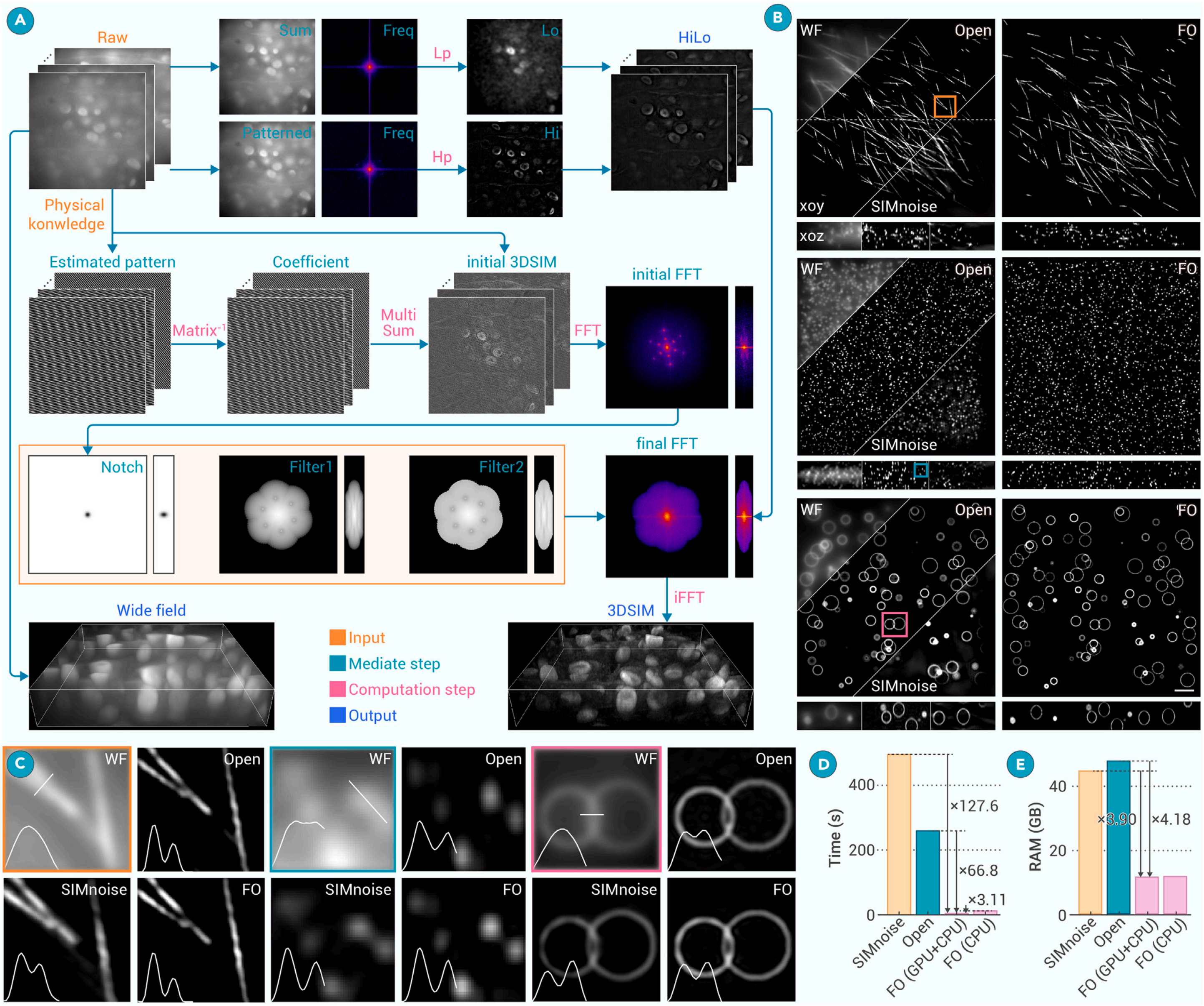

Figure 1. Principles and performance of FO-3DSIM (A) The algorithm flow of FO-3DSIM when reconstructing the hydrilla verticillate sample. Orange, green, pink, and blue represent input, mediate step, computation step, and output. (B) Reconstruction of simulated line, point, and circle-shaped samples using WF, Open-3DSIM, SIMnoise, and FO-3DSIM with the comparisons. (C) The magnified zones in (B) and the corresponding profiles. (D) The reconstruction time of samples in (B), where the size of the image is 512 × 512 × 41 with (E) the RAM used during the reconstruction. The FO-3DSIM is written with CPU + GPU and CPU only for comparison. Axial scale bar, 4 μm. Lateral scale, 41 layers, 125 nm per layer.

Biological samples

The mouse kidney sections are purchased from Thermo Fisher (F24630). The actin/mitochondria/tubulin of Cos-7 cells are purchased from GATTA (GATTA-Cells 4C). The black algae leaves and veins in oleander leaves are purchased from Weike Kejiao (China).

3DSIM imaging

We use four different 3DSIM systems to acquire 3DSIM data, as shown in Table S1. DMD-3DSIM is our home-built 3DSIM system using a digital micro-mirror device in conjunction with an electro-optic modulator. DMD-3DSIM uses a 561 nm excitation laser (MGL-FN-561-200mW, Changchun New Industries Optoelectronics Technology) with objective lens (OBJ, CFI Apochromat TIRF 100XC 1.49NA oil, Nikon). We also obtain data based on the commercial OMX-SIM system (DeltaVision OMX SR, GE) using an oil immersion objective (Olympus, ×60 1.4 numerical aperture [NA]) and a commercial N-SIM2 system (Nikon) using an oil immersion objective (CFI Apochromat, ×100 1.49 NA). OMX is used to image live ER and actin. N-SIM2 is used in large FOV imaging. We lastly use the commercial Airy Polar-SIM system (Airy Technology, China) for multi-color and polarization imaging. Airy Polar-SIM is equipped with 405, 488, 561, and 640 nm channels with a 100× oil-immersion objective from Nikon.

Signal-to-background ratio

We normalize the signal distribution from the original image $I(x, y)$ to the normalized image $I'(x,y)$:

$$SBR(\mathrm{dB}) = 10\,lg\frac{ave[I'(x,y)]_{signal}}{ave[I'(x,y)]_{background}}$$

where $ave[\cdot]$ is the average gray value of the region of interest of signal or background. For fair comparisons, we choose the same area of signal or background using different algorithms.

Signal-to-noise ratio

Signal-to-noise ratio: the part with signal and the part without signal to solve its mean value and variance, and use the following formula to solve signal-to-noise ratio (SNR) (dB):

$$SNR(\mathrm{dB}) = 10\,lg\frac{ave[I(x,y)]_{signal} - ave[I(x,y)]_{noise}}{std[I(x,y)]_{noise}}$$

where $ave[\cdot]$ and $std[\cdot]$ are the average gray value and standard deviation of the region of interest. For fair comparisons, we choose the same area of signal or noise using different algorithms.

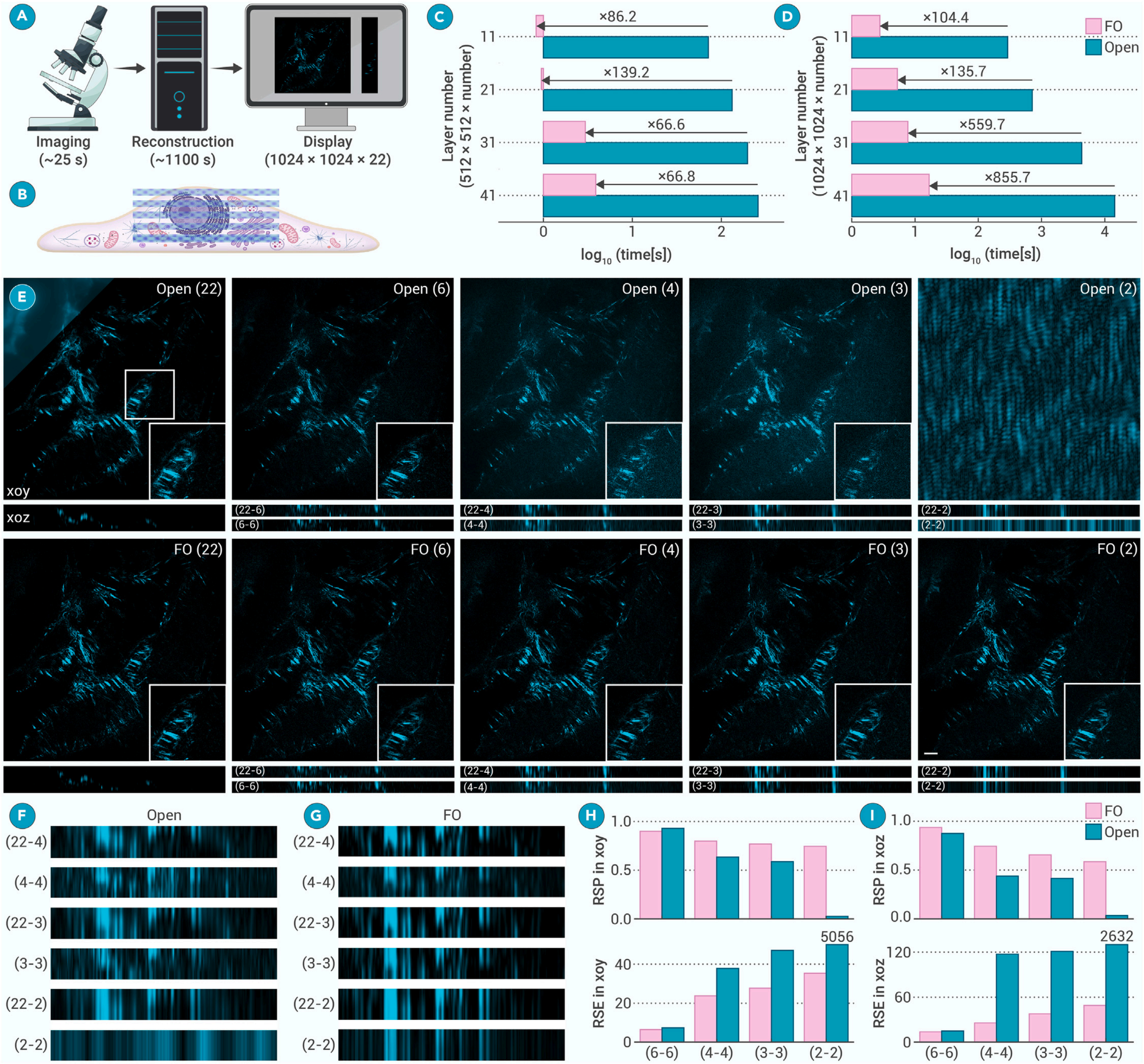

Figure 2. FO-3DSIM increases the reconstruction speed and reconstructs well within limited layers (A) Typical 3DSIM imaging steps including imaging, reconstruction, and display using Open-3DSIM as an example with 1,024 × 1,024 × 22 image size. (B) Sketch map of the layer-by-layer scanning of the whole cell. The $\log_{10}$(time[s]) of reconstruction by Open-3DSIM and FO-3DSIM with image size of (C) 512 × 512 × 11, 512 × 512 × 21, 512 × 512 × 31, 512 × 512 × 41, and (D) 1,024 × 1,024 × 11, 1,024 × 1,024 × 21, 1,024 × 1,024 × 31, 1,024 × 1,024 × 41. (E) Reconstruction results using different *z* layers by Open-3DSIM and FO-3DSIM, where Open (2) means Open-3DSIM using two layers of 3DSIM image. The right bottom part is the magnified zone, where (22-4) means reconstruct using 22 layers and choosing 4 layers for display, (4-4) means reconstruct using 4 layers and choosing 4 layers for display. (F) The magnified *xoz* plane of (F) Open-3DSIM and (G) FO-3DSIM. The RSP and RSE in (H) *xoy* and (I) *xoz* planes using Open-3DSIM and FO-3DSIM. Scale bar, 4 μm.

Computation condition

The time calculation uses a Linux computer (Linux 5.4.0, Intel i9-10900X at 3.70 GHz, NVIDIA GTX 3090, RAM 64 GB, MATLAB R2017). We also test it successfully using a Windows computer (Windows 11, Intel i7-12700, NVIDIA GTX 1650, RAM 64 GB, MATLAB R2021b). The calculation time uses tic and toc in MATLAB, and the RAM occupied is calculated using the task manager.

RESULTS

Principle of FO-3DSIM

Traditional 3DSIM algorithms usually follow these steps[16,19]: the raw 3DSIM data are converted to the frequency domain and the illumination parameters are calculated.[11,20] Then the 0-th-, ±1-st-, and ±2-nd-order components are separated with a phase-separation matrix and shifted to their corresponding frequency place. Various notch filters or optimization filters are then applied, followed by conversion to the spatial domain. The detailed process can be seen in Note S1. Different from frequency reconstruction, we develop FO-3DSIM based on the spatial domain as shown in Figure 1A. The initial super-resolution image $SR_{0,\theta}(r,z)$ in one angle θ at the position (r,z) can be expressed as the sum of raw data with different phases:

$$SR_{0,\theta}(r,z) = \sum_{i=1}^{5} o_{\theta,i}(r,z)\cdot D_{\theta,\phi}(r,z) \qquad \text{(Equation 1)}$$

where $o_{\theta,i}(r,z)$, i = 1, 2, 3, 4, and 5, are the coefficients, i means the i-th phase ϕ. and $D_{\theta,\phi}(r,z)$ is the emission distribution in phase ϕ. Without loss of generality,

we assume the phase shift $\omega r_i'$ is $0, \frac{2}{5}\pi, \frac{4}{5}\pi, \frac{6}{5}\pi, \frac{8}{5}\pi$. After we subtract the 0-th components in $SR_{0,\theta}(r,z)$, we can resolve the corrected coefficients $o_{\theta,i}(r,z)$ according to the following equations as shown in Note S2:

$$\begin{bmatrix} c_{\theta,1}(r,z) \\ c_{\theta,2}(r,z) \\ c_{\theta,3}(r,z) \\ c_{\theta,4}(r,z) \\ c_{\theta,5}(r,z) \end{bmatrix} \begin{bmatrix} 0 & 0 & 0 & 0 & 0 \\ \cos 0 & \cos\frac{2}{5}\pi & \cos\frac{4}{5}\pi & \cos\frac{6}{5}\pi & \cos\frac{8}{5}\pi \\ \sin 0 & \sin\frac{2}{5}\pi & \sin\frac{4}{5}\pi & \sin\frac{6}{5}\pi & \sin\frac{8}{5}\pi \\ \cos 0 & \cos\frac{4}{5}\pi & \cos\frac{8}{5}\pi & \cos\frac{12}{5}\pi & \cos\frac{16}{5}\pi \\ \sin 0 & \sin\frac{4}{5}\pi & \sin\frac{8}{5}\pi & \sin\frac{12}{5}\pi & \sin\frac{16}{5}\pi \end{bmatrix} = \begin{bmatrix} \frac{1}{I_0} \\ \frac{\cos(\omega r+\phi)}{a_1 I_0} \\ \frac{\sin(\omega r+\phi)}{a_1 I_0} \\ \frac{\cos(2\omega r+2\phi)}{a_2 I_0} \\ \frac{\sin(2\omega r+2\phi)}{a_2 I_0} \end{bmatrix} \quad \text{(Equation 2)}$$

So, the super-resolution image $SR_{0,-}(r,z)$ (minus the 0-th component) can be expressed as:

$$SR_{0,-}(r,z) = \sum_{\theta=1}^{3}\sum_{i=1}^{5} c_{\theta,i}(r,z)\cdot D_{\theta,i}(r,z) \quad \text{(Equation 3)}$$

Then, we convert the super-resolution image into the frequency domain and use the notch filter and spectrum filter to optimize the reconstruction. The optimized super-resolution image is $SR_1(r,z)$. Note that we subtract the 0-th component to suppress the defocused background. Because 3DSIM has lower harmonics (~1.5 spread spectrum) compared with 2DSIM, introducing HiLo into 3DSIM fully corresponds to the requirement of HiLo.[21]

$$OS(r,z) = \sum_{\theta=1}^{3}\left\{Lo[D_{\theta,1}(r,z)] + Hi\left[\sum_{i=1}^{5} D_{\theta,i}(r,z)/5\right]\right\}/3 \quad \text{(Equation 4)}$$

where $Lo[\cdot]$ and $Hi[\cdot]$ are low-pass and high-pass filter, respectively, and we average the three angles of illumination to avoid light intensity difference in three angles. At last, the final super-resolution image $SR_2(r,z)$ can be expressed as:

$$SR_2(r,z) = SR_1(r,z) + OS(r,z) \quad \text{(Equation 5)}$$

The overall flow is shown in Figure 1A. Because the spectrum filters and physical knowledge (wavelength, NA, and illumination parameters) are only related to the microscope, we set them as prior knowledge. The reconstruction flow includes the HiLo reconstruction, coefficient calculation, summarization, and spectrum optimization. The simulated results are shown in Figure 1B using simulated line-, point-, and circle-shaped 3D graphics, where the comparison includes WF, Open-3DSIM,[16] and SIMnoise,[19] and the magnified zones are shown in Figure 1C. It can be seen that the unresolved 3D line, point, and circle shape in WF can be clearly distinguished by FO-3DSIM, demonstrating that FO-3DSIM has the same improvement in the *xyz* directions as Open-3DSIM and SIMnoise do. Image decorrelation analysis for the quantified resolutions of WF, FO-3DSIM, Open-3DSIM, and SIMnoise are 237.25, 130.66, 134.20, and 141.67 nm, respectively, using the point-shaped simulation.[22] This showcases the near double resolution improvement than WF without any compromise of resolution. In Note S3, we demonstrate that the reconstruction principle of FO-3DSIM is essentially the same as traditional 3DSIM despite the simplified flow, without any compromise of resolution and fidelity. It is noteworthy that introducing HiLo is used to suppress the defocused background not to improve the axial resolution. As a counterpart, we calculate the core reconstruction time and RAM used in the core reconstruction part using FO-3DSIM (which is accelerated by GPU), Open-3DSIM, and SIMnoise. FO-3DSIM has a 127.6 and 88.2 times reconstruction speed and 3.90 and 4.18 times RAM reduction compared with Open-3DSIM and SIMnoise in an image size of 512 × 512 × 41 in Figures 1D and 1E. Furthermore, we rewrite FO-3DSIM into CPU calculation only, taking about 12.1s to reconstruct, which is still far less than Open-3DSIM (259s), proving that the acceleration lies in its inherent principle rather than GPU acceleration only. To better compare the reconstruction flow, we also list the time and computational complexity of immediate steps in Open-3DSIM and FO-3DSIM in Figure S1. SIMnoise takes a more time-consuming method with many optimization operations, and it uses ROM and RAM to store data. So, it is listed relatively separately in Figure S1. Through Note S3, we demonstrate that FO-3DSIM has the same spectrum after the reconstruction, which is the origin of the twice axial resolution improvement as shown in Figures 1B and 1C.

FO-3DSIM demonstrates superior performance

In the last section, we demonstrated that FO-3DSIM has a high-quality reconstruction performance and, here, we show its superior performance compared with other algorithms. A typical 3DSIM imaging process shown in Figure 2A contains the imaging, reconstruction, and display. When imaging a sample with a 1,024 × 1,024 × 22 image size, the imaging time in a commercial system is about 25 s. However, users should wait for about 1,100 s in reconstruction, using Open-3DSIM, for example, and then the super-resolution image can be displayed. This process greatly increases the difficulty of large-field and real-time observation of samples, hindering feedback capture of cellular states. The *z* layer scanning process is shown in Figure 2B; traditional 3DSIM needs more than six layers (about 0.75 μm) to reconstruct a high-quality image. But, when imaging cells where 2DSIM will cause defocused artifacts and the thickness is less than 0.75 μm, traditional 3DSIM scanning more than six layers cannot provide additional information but will cause a decrease in temporal resolution and more optical damage.

Here, we demonstrate that FO-3DSIM can resolve the problems mentioned above. Because Open-3DSIM has about 2-fold speed improvement compared with SIMnoise in Figure 1D,[19] we compared FO-3DSIM and Open-3DSIM in Figures 2C and 2D using different image sizes. We can find that FO-3DSIM has an improvement compared with Open-3DSIM by a minimum of 66.6 times and a maximum of 855.7 times. Taking a 1,024 × 1,024 × 41 image as an example, the core reconstruction time of Open-3DSIM is about 4 h to reconstruct, but that FO-3DSIM is only 16.72 s. This is because of the RAM usage. With the increase in image size, the RAM usage has exponential growth, but the maximum RAM used in our experiment is 64 GB fixed. So, when running Open-3DSIM, the RAM is in a fully occupied state, which will largely lag the reconstruction time. This reflects the fast reconstruction capability of FO-3DSIM and can decrease the reconstruction time less than the imaging time, revealing the potential to realize real-time 3D imaging. It is noteworthy that the parameter estimation is used as prior knowledge for reconstruction. Because the parameter estimations for 3DSIM and single-layer 3DSIM are the same, the time for reconstructing 512 × 512 × *N* and 1,024 × 1,024 × *N* is 9.76 and 34.12 s, respectively.

Then, we showcase that FO-3DSIM can reconstruct well even with 2 *z* layers in Figure 2E, with the reference of reconstruction under 22 *z* layers. This is because the 3D Weiner filter in the traditional method will be distorted when the image layer is small and OTF is not accurate. However, FO-3DSIM with the 0-th component removed previously will reduce the distortion introduced from an inaccurate filter, thus enabling reconstruction with higher fidelity under

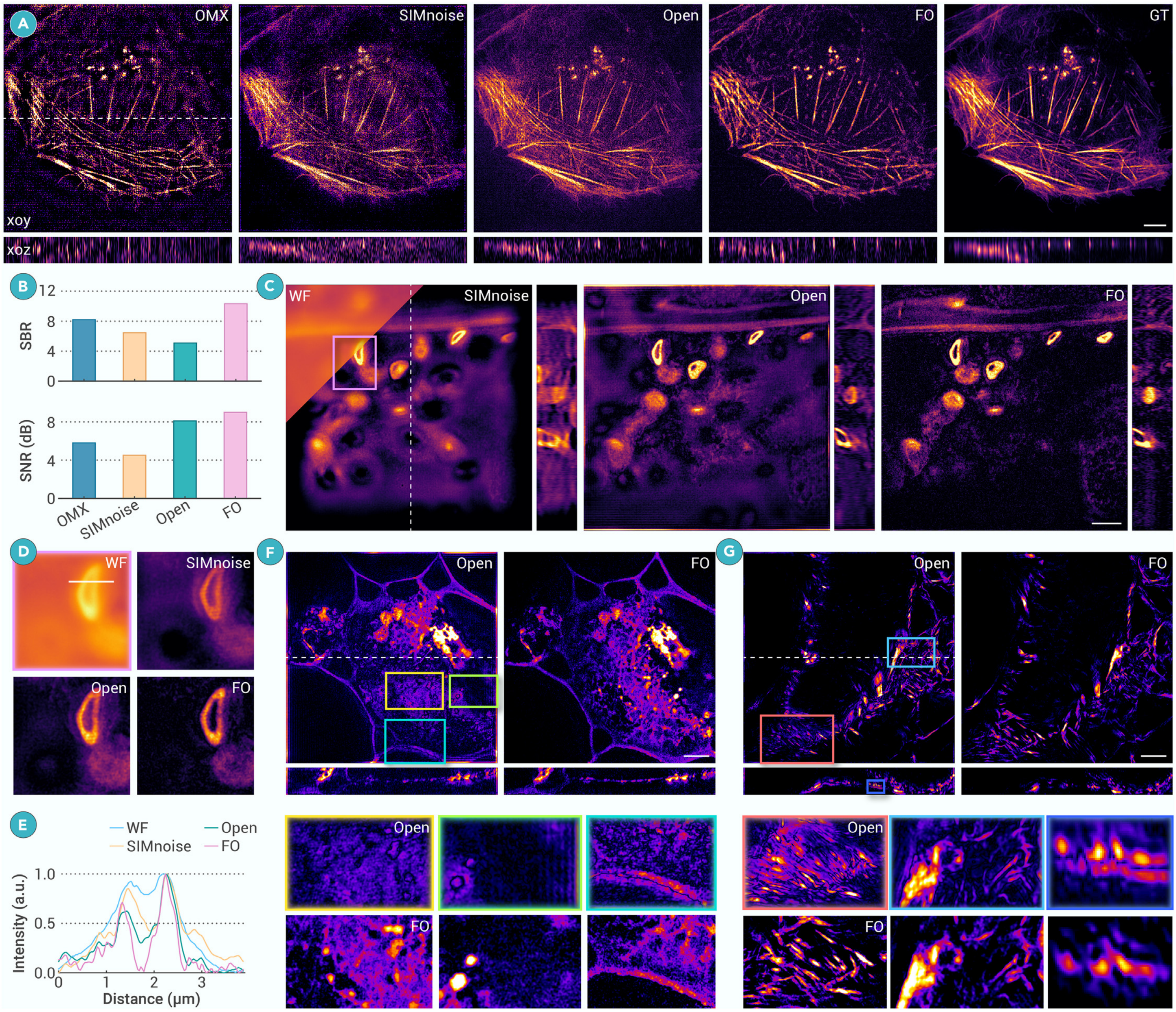

Figure 3. FO-3DSIM has superior reconstruction performance compared with other algorithms (A) The reconstruction results of OMX, SIMnoise, Open-3DSIM, and FO-3DSIM under extremely low illumination dose using actin filament. The right part is the GT image under a high illumination dose. (B) Quantitative evaluation of SBR and SNR in (A). (C) Comparison between WF, SIMnoise, Open-3DSIM, and FO-3DSIM when imaging a thick sample using black algae leaves with (D) the magnified zone and (E) its intensity profile along the white line. The comparison between Open-3DSIM and FO-3DSIM when imaging thick samples using (F) veins in oleander leaves and (G) actin filament in mouse kidney section and their magnified zones. Lateral scale bar, 4 μm. Axial scales, (A) 13 layers, (C) 30 layers, (F) 44 layers, (G) 37 layers, 125 nm per layer.

limited layers. Open-3DSIM using 2 layers cannot reconstruct at all, and results using 3, 4, and 5 layers will bring many artifacts, as shown as the magnified zones in the right bottom parts. The magnified *xoz* planes in Figure 2F using Open-3DSIM and Figure 2G using FO-3DSIM and the quantitative results of RSP and RSE in *xoy* and *xoz* planes demonstrates the superior performance of FO-3DSIM under limited *z* layers in Figures 2H and 2I. It is noteworthy that single-layer 3DSIM cannot bring resolution improvement in the *xoz* plane. Therefore, FO-3DSIM ensures an enhancement in the *z* axis resolution with very few *z* layers, bridging the gap between single-layer 3DSIM and multi-layer 3DSIM.

Reconstructing under low SNR and severe defocused background is a typical challenging problem in 3DSIM. In Open-3DSIM, we use a two-step filter and notch filter to suppress noise and background, respectively. Here, we also introduce the two-step filter into FO-3DSIM to ensure the performance under low SNR. Using the open-source data of actin filament under extremely low illumination conditions, we compare OMX, SIMnoise, Open-3DSIM, FO-3DSIM, and GT-3DSIM in Figure 3A. The quantitative evaluation of signal-to-background ratio (SBR) and SNR of those algorithms is shown in Figure 3B. From both visual and quantitative views, FO-3DSIM is closer to GT images and possesses a relatively higher SBR and SNR. It can be seen that FO-3DSIM fully inherits the advantages of Open-3DSIM in reconstruction under low SNR conditions.

To show the advantage of combing 3DSIM and OS-SIM, we do the comparison before and after introducing OS-SIM into 3DSIM, as shown in Figure S2, finding that OS-SIM can effectively improve the optical sectioning performance of FO-3DSIM. Then, we use the black algae leaves with overwhelmed backgrounds and compare the performance of SIMnoise, Open-3DSIM, and FO-3DSIM in Figure 3C. The magnified zone shown in Figure 3D shows that FO-3DSIM can observe the hollow structure of black algae leaves more clearly. We can find that FO-3DSIM has a better optical sectioning capability, and this can be of help to distinguish the hollow structure clearly with the removal of the background in the hollow center in Figure 3E. Open-3DSIM uses a notch filter to suppress the background, but this process will normally introduce artifacts as shown in Figures 3F and 3G using veins in oleander leaves and actin filament in mouse kidney section as examples.

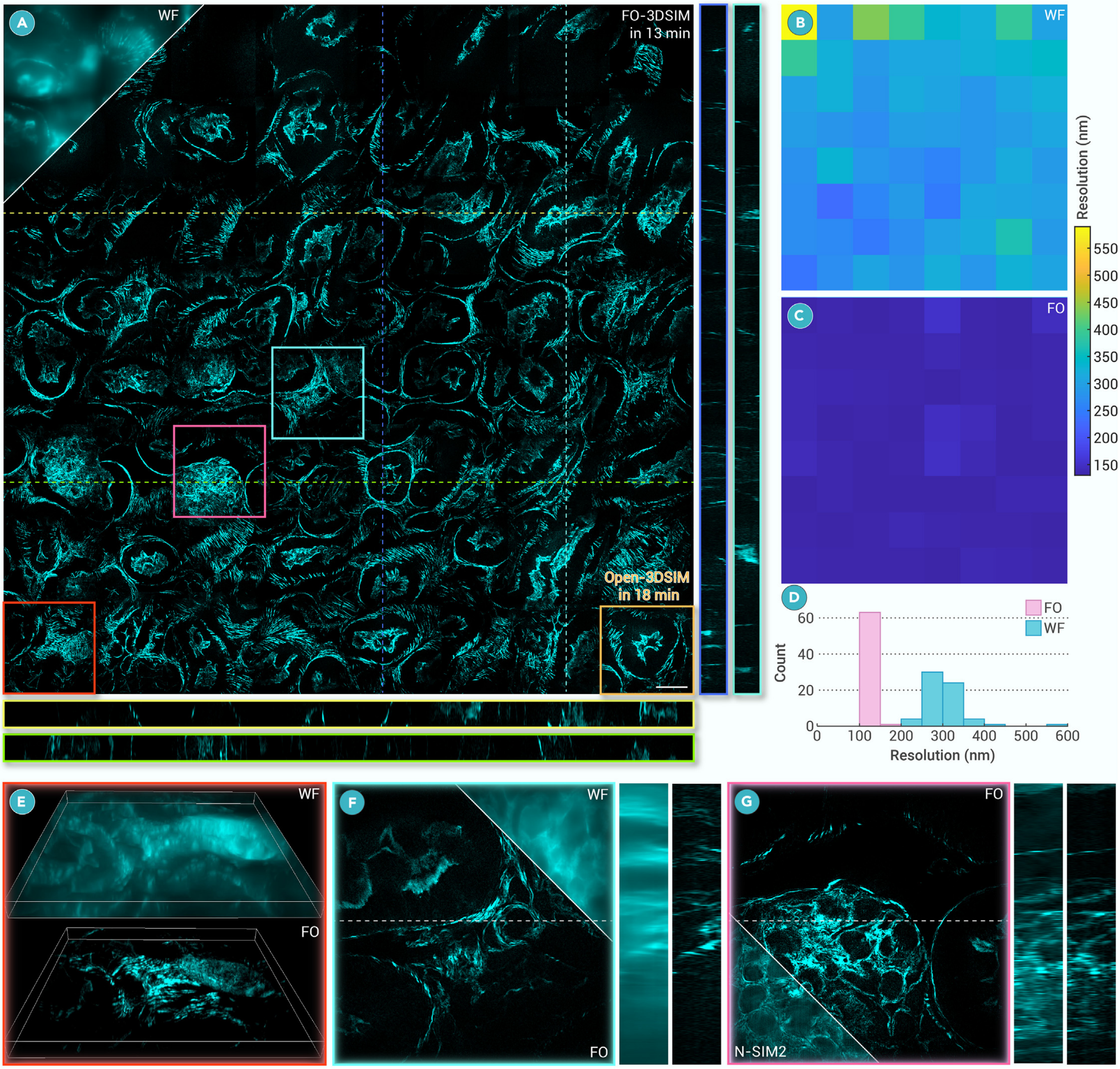

Figure 4. FO-3DSIM assists in 3D large FOV super-resolution imaging (A) The maximum intensity projection of 3D large FOV reconstruction of actin filament in mouse kidney section in *xoy*, *xoz*, and *yoz* planes within 13 min using FO-3DSIM. The yellow box denotes the reconstruction of Open-3DSIM within 18 min. (B) The resolution map of maximum intensity projection of WF in (A) using decorrelation analysis with the corresponding comparison of (C) 3DSIM dividing the image into 8 × 8 parts. (D) The statistical histograms of WF and FO-3DSIM resolutions. (E) Local comparison of 3D images with WF and FO-3DSIM. (F) Local comparison of 3D images with WF and 3DSIM in *xoy* and *xoz* plane. (G) Comparison between Nikon's N-SIM2 software and FO-3DSIM in a local zone. Lateral scale bar, 20 μm. Axial scale: 22 layers, 125 nm per layer.

The structure of veins disappears in Open-3DSIM, but FO-3DSIM can recover it to a great extent. What is more, FO-3DSIM can recover the edge information with no pattern-shaped artifacts, as shown in the magnified zone in Figure 3F. Similarly, FO-3DSIM can eliminate the artifacts that exist in Open-3DSIM in Figure 3G in both *xoy* and *xoz* planes. As a result, FO-3DSIM can reconstruct well under overwhelming defocused backgrounds by introducing the operation of optical sectioning capability. It is noteworthy that combining OS-SIM with 3DSIM is a common method that not only can be applied to FO-3DSIM but can further reduce the artifacts and improve the reconstruction using traditional 3DSIM method such as Open-3DSIM in Figure S3.

FO-3DSIM assists 3D large FOV imaging

Benefiting from the fast reconstruction and optical sectioning performance of FO-3DSIM, we use it to conduct 3D super-resolution imaging of large FOV. Although 3DSIM is suitable for scanning FOV imaging, the reconstruction burden is very high. Here, we conduct a 4,530 × 4,530 × 2.75 μm imaging combining 8 × 8 zones (every zone means 1,024 × 1,024 × 22 pixel sizes with ~17% overlap) of actin filament in mouse kidney tissue section in Figure 4A and Video S1. The imaging time using Nikon's N-SIM2 system is 23 min and FO-3DSIM can reconstruct the whole FOV in 13 min, which is almost twice less than the imaging time. Using Open-3DSIM can only reconstruct 1/64 of the whole FOV in 18 min, meaning that using Open-3DSIM to reconstruct all of the FOV needs about

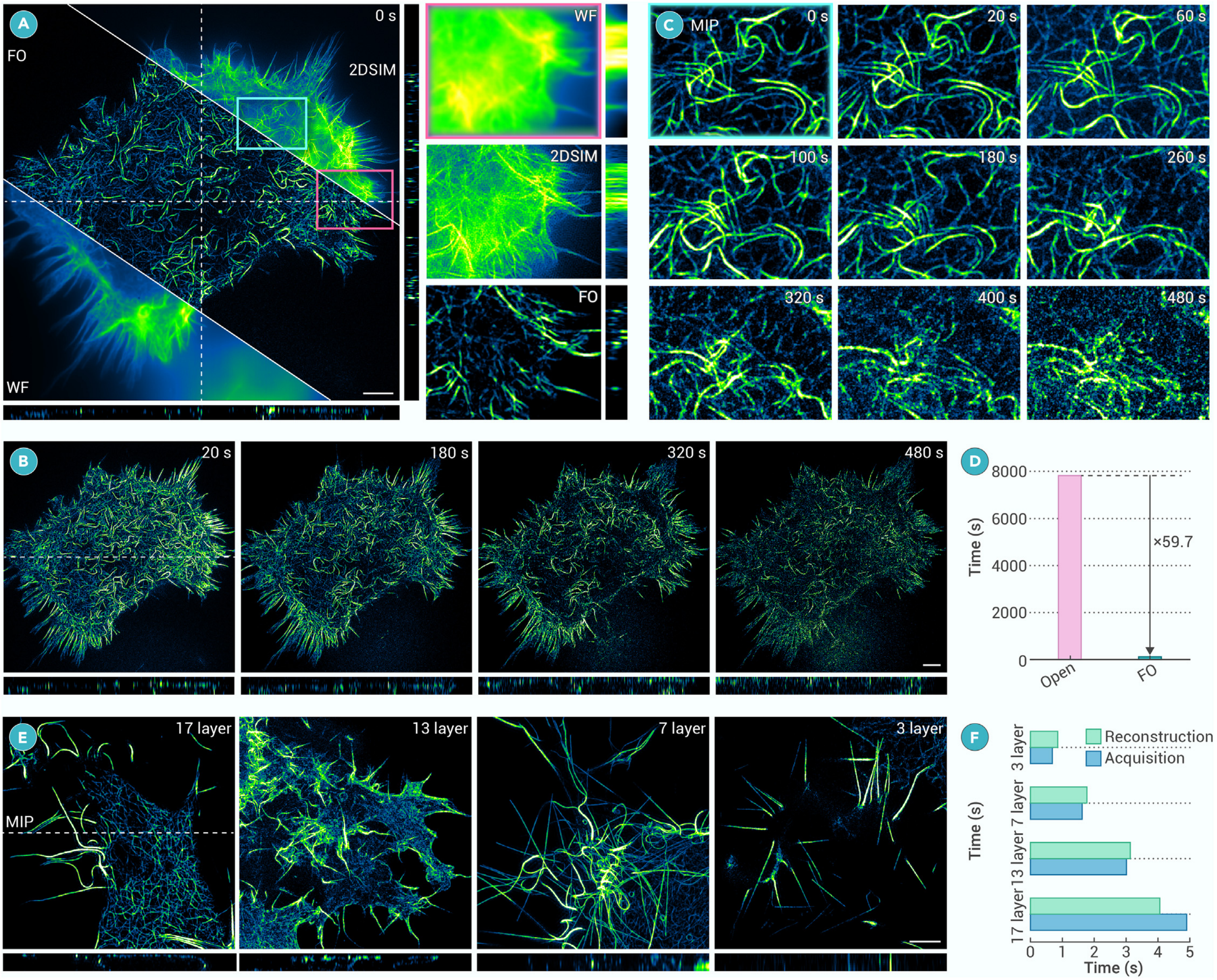

Figure 5. FO-3DSIM prominently reduces the reconstruction time of huge datasets and achieves near real-time reconstruction (A) Comparison between WF, 2DSIM, and FO-3DSIM when imaging living actin filament in cos-7 cells with the magnified zones in the left part. (B) Whole FOV of 3D time-lapse imaging of actin filament with the (C) magnified zones. There are a total of 25 frames, 1,024 × 1,024 × 7 pixels in each frame. (D) The whole reconstruction time of Open-3DSIM and FO-3DSIM. (E) The reconstruction of living actin filament under 3, 7, 13, and 17 layers, along with the comparison between reconstruction time and acquisition time using the OMX system under (F) 17, 13, 7, and 3 layers. Lateral scale bar, 4 μm. Axial scale bar, (A) 7 layers, 125 nm per layer.

19.2 h. To evaluate the resolution, we use image decorrelation to calculate the resolution in the 64 zones of the whole FOV in Figures 4B and 4C, finding that FO-3DSIM can achieve the double resolution improvement from ~280 to ~140 nm with minimum artifacts, as illustrated as the statistical results in Figure 4D. Actin in the mouse kidney section has overwhelmed backgrounds in WF images, and the actin structure can hardly be distinguished, as illustrated in the 3D view in Figures 4E and 4F, with a clear improvement of resolution in the *xoz* plane. Finally, we compare the reconstruction of N-SIM2 with FO-3DSIM in Figure 4G, finding that FO-3DSIM can better remove the defocused background due to its optical sectioning capability.

FO-3DSIM assists near real-time imaging and limited-layer reconstruction

Real-time is another grand challenge in 3DSIM because the reconstruction time is huge. When imaging samples with certain thickness, WF and 2DSIM cannot reconstruct well because of the defocused background and limited axial resolution. As shown in Figure 5A, OMX-2DSIM will introduce artifacts and its axial resolution is the same as WF, while 3DSIM can observe the 3D structure of actin filament clearly. Although there are other advanced 2DSIM algorithms, such as BF-SIM and HiFi-SIM,[11,17] 3DSIM has irreplaceable advantages for thick sample imaging, as shown in Figure S4. The time-lapse imaging of the actin filament is shown in Figure 5B and the maximum intensity projection view of the local zone is shown in Figure 5C using 7-layer imaging. We can find that, because of the relatively more *z* layers, results show optical damage to the cell, and the results of 480 s show unideal reconstruction with photobleaching of fluorescent dyes. After acquiring 25 frames of 1,024 × 1,024 × 7 images, reconstruction will take a long time, as shown in Figure 5D. The reconstruction time of traditional Open-3DSIM takes about 8,000 s, while FO-3DSIM only takes 131 s, showing a 59.7-fold improvement. What is more, we compare the acquisition time and reconstruction time using different *z* layers when imaging actin filament in Figure 5E, and the comparisons are shown in Figure 5F. We use the fastest 3DSIM commercial system GE | OMX, as we know, for comparison. FO-3DSIM can reconstruct well under 3, 7, 13, and 17 layers when the image size is 1,024 × 1,024. FO-3DSIM adopts a sequence of acquisition-reconstruction-acquisition-reconstruction, which is expected to cooperate with hardware implementation to achieve near real-time reconstruction and observation. This is different from traditional 3DSIM as shown in Figure 2A, which provides great convenience for biological users and enables real-time super-resolution observation of subcellular movements and interactions.

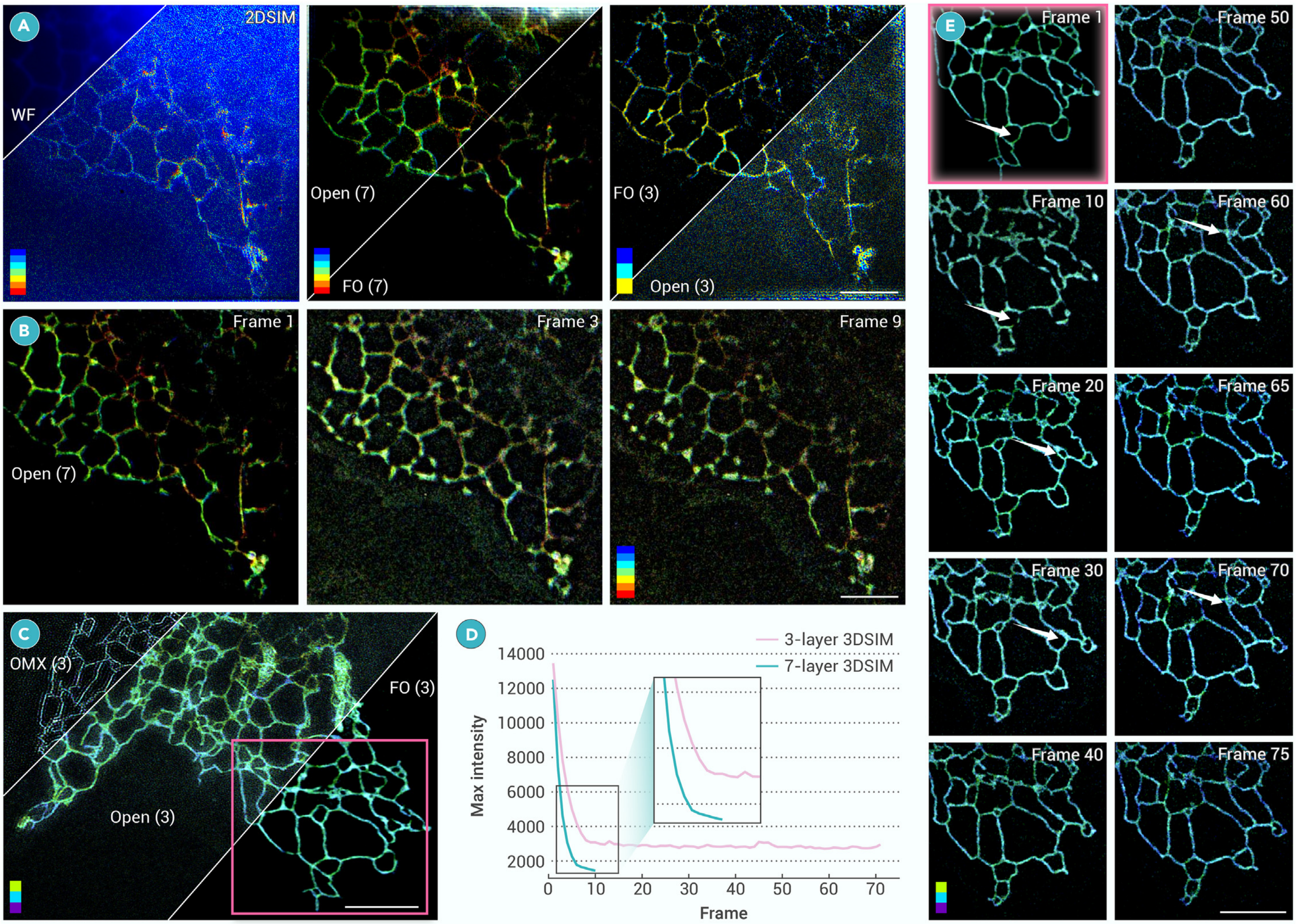

Figure 6. Limited-layer reconstruction produces lower photodamage (A) Comparison between WF, 2DSIM, Open-3DSIM, and FO-3DSIM using 7-layer and 3-layer reconstruction of live ER, where Open(7) means the 7-layer reconstruction using Open-3DSIM, and FO(7) means the 7-layer reconstruction using FO-3DSIM. (B) Time-lapse imaging for 7-layer 3DSIM, demonstrating severe photon damage to the sample. (C) Time-lapse 3-layer imaging of ER with the comparison between OMX-3DSIM, Open-3DSIM, and FO-3DSIM (D) The gradual decrease of image intensity using 7-layer 3DSIM and 3-layer 3DSIM with the change of frame number, where per frame means 4 s. (E) Magnified zone of 3-layer 3DSIM imaging reveals the motion of the ER. Scale bar, 4 μm.

Finally, optical damage is another primary factor limiting the development of 3DSIM.[23] However, 3DSIM is more suitable than 2DSIM when imaging samples with defocused backgrounds, as shown in Figure 6A. 2DSIM and WF cannot distinguish the ER tubes because of the defocused background, while Open-3DSIM and FO-3DSIM can reconstruct well using a 7-layer situation. However, ER tubes are thin but with backgrounds, and there is no need to image a 7-layer FOV. Three layers are sufficient to recover most of the sample information in Figure 6A. In this case, traditional 3DSIM reconstruction based on frequency operation fails with many artifacts, but FO-3DSIM can reconstruct well. Furthermore, we compared long-term imaging using 7-layer reconstruction versus 3-layer reconstruction. In 7-layer imaging, we used 7 layers with 105 frames per volume for reconstruction, but 45 frames per volume were used in 3-layer imaging as a counterpart. We found that the ER underwent severe photobleaching after 6 frames, making reconstructing almost impossible, as shown in Figure 6B. As shown in Figure 6C, the 3-layer 3DSIM of the OMX and Open-3DSIM failed to reconstruct, whereas FO-3DSIM demonstrated superior reconstruction performance. The fluorescence bleaching curve for 3-layer reconstruction with 45 frames per volume is shown in Figure 6D, where 3-layer reconstruction maintained reasonable fluorescence intensity even after 75 frames and can be reconstructed well. From a quantitative perspective, the phototoxicity of 3-layer 3DSIM is more than two times lower than that of 7-layer 3DSIM, allowing cells to recover during imaging intervals. Therefore, it enables more than seven times the imaging duration over the traditional methods in the live-cell imaging. Taking advantage of 3-layer reconstruction, we also observed ER motion phenomena with almost no artifacts and defocused backgrounds in Figure 6E. As indicated by the white arrows, the endoplasmic reticulum undergoes fragmentation and reorganization, transitioning from ring-shaped to linear structures, and from punctate to ring-shaped structures. Therefore, FO-3DSIM in 3-layer reconstruction does not excessively affect the cellular state as shown in Video S2.

DISCUSSION

Furthermore, we demonstrate the capability of FO-3DSIM to achieve multi-color imaging (Figure S5) and polarization imaging (Figure S6) with no introduction of additional hardware. It fully inherits the capabilities of Open-3DSIM in 6D imaging including *xyz* super-resolution, multi-color, long-term, and fluorescence dipole imaging, thereby contributing to a more comprehensive understanding of biological phenomena. Its advantage in reconstruction with fewer layers significantly reduces the phototoxicity and photobleaching of traditional 3DSIM, extends the imaging duration for long-term studies, and enhances the fidelity and capability of thick-sample reconstruction by improving the physical suppression of defocused backgrounds.

We choose the traditional COR method for parameter estimation and, because of the relatively long time, the parameter estimation part is set as the prior knowledge of reconstruction. During the live actin or ER and high-throughput imaging in Figures 4, 5, and 6, the parameter for each sample remains unchanged and it works well.[13,14] However, we still hope that a faster parameter estimation method can also be applied to FO-3DSIM seamlessly to achieve real-time parameter adjustment. An example is PCA-SIM, which

optimizes the parameter to realize estimation in every frame to realize real-time 2DSIM.[20]

Previously, we introduced the DMD-3DSIM optical system,[24] which offers high-speed and self-polarizing advantages. However, due to limitations in live-cell workstations, the relatively low readout time of sCMOS, and the interaction between software and hardware, we utilized the fastest available commercial system, OMX, to demonstrate the real-time imaging potential of FO-3DSIM, without achieving true real-time 3DSIM observation. However, FO-3DSIM can match reconstruction time with acquisition time to the same level. To further increase the real-time performance, converting MATLAB into C/Java may be a good choice for the language advantage on processing speed. We previously commercialized Polar-SIM through Airy Technologies, in which by rewriting the algorithm into C and integrating CUDA parallel computing, it granted the Polar-SIM system with high-speed reconstruction. We are looking forward to the deployment of FO-3DSIM in the near future, to advance the realization of real-time 3DSIM reconstruction.

CONCLUSION

Since the development of Open-3DSIM and DMD-3DSIM, we are pleased to witness the advancement of various hardware and software implementations based on this platform, driving the application of 6D modalities of 3DSIM in biology. As a fundamental and advanced algorithm, FO-3DSIM not only significantly enhances reconstruction speed and the capability for reconstruction of thin-layered/thick samples, but also integrates seamlessly with various deconvolution, denoising, and background removal algorithms, thereby further improving the resolution, SNR, and other performance metrics of 3DSIM. Moreover, it can also be combined with z axis resolution enhancement techniques such as 4B-3DSIM,[25] adaptive optics methods such as AO-3DSIM,[26] or parallel acquisition-readout methods such as PAR-SIM.[6] Thus, we hope it can ultimately provide rapid and high-quality 3DSIM reconstruction for a wide range of commercial and research-grade 3DSIM instruments.

DATA AND CODE AVAILABILITY

The data and code in this manuscript are provided in github: https://github.com/Caoruijie/FO-3DSIM.

REFERENCES

1. Gustafsson, M.G. (2000). Surpassing the lateral resolution limit by a factor of two using structured illumination microscopy. J. Microsc. ***198***: 82–87. https://doi.org/10.1046/j.1365-2818.2000.00710.x.
2. Gustafsson, M.G. (1999). Extended resolution fluorescence microscopy. Curr. Opin. Struct. Biol. ***9***: 627–634. https://doi.org/10.1016/S0959-440X(99)00016-0.
3. Huang, X., Fan, J., Li, L., et al. (2018). Fast, long-term, super-resolution imaging with Hessian structured illumination microscopy. Nat. Biotechnol. ***36***: 451–459. https://doi.org/10.1038/nbt.4115.
4. Guo, Y., Li, D., Zhang, S., et al. (2018). Visualizing Intracellular Organelle and Cytoskeletal Interactions at Nanoscale Resolution on Millisecond Timescales. Cell ***175***(10): 1430–1442.e17. https://doi.org/10.1016/j.cell.2018.09.057.
5. Ren, W., Guan, M., Liang, Q., et al. (2024). Expanding super-resolution imaging versatility in organisms with multi-confocal image scanning microscopy. Natl. Sci. Rev. ***11***: nwae303. https://doi.org/10.1093/nsr/nwae303.
6. Xu, X., Wang, W., Qiao, L., et al. (2024). Ultra-high spatio-temporal resolution imaging with parallel acquisition-readout structured illumination microscopy (PAR-SIM). Light Sci. Appl. ***13***: 125. https://doi.org/10.1038/s41377-024-01464-8.
7. Gustafsson, M.G.L., Shao, L., Carlton, P.M., et al. (2008). Three-dimensional resolution doubling in wide-field fluorescence microscopy by structured illumination. Biophys. J. ***94***: 4957–4970. https://doi.org/10.1529/biophysj.107.120345.
8. Chen, X., Zhong, S., Hou, Y., et al. (2023). Superresolution structured illumination microscopy reconstruction algorithms: a review. Light Sci. Appl. ***12***: 172. https://doi.org/10.1038/s41377-023-01204-4.
9. Zhao, W., Zhao, S., Li, L., et al. (2022). Sparse deconvolution improves the resolution of live-cell super-resolution fluorescence microscopy. Nat. Biotechnol. ***40***: 606–617. https://doi.org/10.1038/s41587-021-01092-2.
10. Müller, M., Mönkemöller, V., Hennig, S., et al. (2016). Open-source image reconstruction of super-resolution structured illumination microscopy data in ImageJ. Nat. Commun. ***7***: 10980. https://doi.org/10.1038/ncomms10980.
11. Wen, G., Li, S., Wang, L., et al. (2021). High-fidelity structured illumination microscopy by point-spread-function engineering. Light Sci. Appl. ***10***: 70. https://doi.org/10.1038/s41377-021-00513-w.
12. Li, D., Shao, L., Chen, B.C., et al. (2015). ADVANCED IMAGING.Extended-resolution structured illumination imaging of endocytic and cytoskeletal dynamics. Science ***349***: aab3500. https://doi.org/10.1126/science.aab3500.
13. Wang, Z., Zhao, T., Cai, Y., et al. (2023). Rapid, artifact-reduced, image reconstruction for super-resolution structured illumination microscopy. Innovation ***4***: 100425. https://doi.org/10.1016/j.xinn.2023.100425.
14. Zhaojun, W., Tianyu, Z., Huiwen, H., et al. (2022). High-speed image reconstruction for optically sectioned, super-resolution structured illumination microscopy. Adv. Photonics ***4***: 026003. https://doi.org/10.1117/1.AP.4.2.026003.
15. Zhao, T., Wang, Z., Cai, Y., et al. (2023). Fast single-layer reconstruction for three-dimensional structured illumination microscopy. Opt Laser. Eng. ***167***: 107606. https://doi.org/10.1016/j.optlaseng.2023.107606.
16. Cao, R., Li, Y., et al. (2023). Open-3DSIM: an open-source three-dimensional structured illumination microscopy reconstruction platform. Nat. Methods ***20***: 1183–1186. https://doi.org/10.1038/s41592-023-01958-0.
17. Mo, Y., Wang, K., Li, L., et al. (2023). Quantitative structured illumination microscopy via a physical model-based background filtering algorithm reveals actin dynamics. Nat. Commun. ***14***: 3089. https://doi.org/10.1038/s41467-023-38808-8.
18. Temma, K., Oketani, R., Kubo, T., et al. (2024). Selective-plane-activation structured illumination microscopy. Nat. Methods ***21***: 889–896. https://doi.org/10.1038/s41592-024-02236-3.
19. Smith, C.S., Slotman, J.A., Schermelleh, L., et al. (2021). Structured illumination microscopy with noise-controlled image reconstructions. Nat. Methods ***18***: 821–828. https://doi.org/10.1038/s41592-021-01167-7.
20. Qian, J., Cao, Y., Bi, Y., et al. (2023). Structured illumination microscopy based on principal component analysis. eLight ***3***: 4. https://doi.org/10.1186/s43593-022-00035-x.
21. Lim, D., Chu, K.K., and Mertz, J. (2008). Wide-field fluorescence sectioning with hybrid speckle and uniform-illumination microscopy. Opt. Lett. ***33***: 1819–1821. https://doi.org/10.1364/OL.33.001819.
22. Descloux, A., Grußmayer, K.S., and Radenovic, A. (2019). Parameter-free image resolution estimation based on decorrelation analysis. Nat. Methods ***16***: 918–924. https://doi.org/10.1038/s41592-019-0515-7.
23. Ren, W., Ge, X., Li, M., et al. (2024). Visualization of cristae and mtDNA interactions via STED nanoscopy using a low saturation power probe. Light Sci. Appl. ***13***: 116. https://doi.org/10.1038/s41377-024-01463-9.
24. Li, Y., Cao, R., Ren, W., et al. (2024). High-speed autopolarization synchronization modulation three-dimensional structured illumination microscopy. Adv. Photon. Nexus ***3***: 016001. https://doi.org/10.1101/2023.12.04.569876.
25. Zhao, W., Zhao, S., Han, Z., et al. (2023). Enhanced detection of fluorescence fluctuations for high-throughput super-resolution imaging. Nat. Photonics ***17***: 806–813. https://doi.org/10.1038/s41566-023-01234-9.
26. Lin, R., Kipreos, E.T., Zhu, J., et al. (2021). Subcellular three-dimensional imaging deep through multicellular thick samples by structured illumination microscopy and adaptive optics. Nat. Commun. ***12***: 3148. https://doi.org/10.1038/s41467-021-23449-6.

ACKNOWLEDGMENTS

This work was supported by the National Key R&D Program of China (2022YFC3401100) and the National Natural Science Foundation of China (624B2009, 62405010, 62335008, 62025501, 92150301, and 62411540238). We thank the National Center for Protein Sciences and the Core Facilities of Life Sciences at Peking University for the GE DeltaVision OMX system. We would like to acknowledge the assistance of SLSTU-Nikon Biological Imaging Center for assistance of using the Nikon N-SIM2 instrument. The funders had no role in study design, data collection and analysis, decision to publish, or preparation of the manuscript.

AUTHOR CONTRIBUTIONS

P.X. and M.L. supervised the project. R.C. and P.X. initiated and conceived the research. R.C. developed the method and experiment. Y.L. constructed the DMD-3DSIM system. W.W. conducted the image montage. X.B. helped with live ER imaging experiments. Y.F. helped with the live actin experiments. D.S., J.S., X.G., B.G., and Z.C. did cell culture. Y.P. provides the computation resource. S.J. helped with the combination of OS-SIM. R.C. drew the figures and videos. R.C., Y.L., M.L., and P.X. wrote the manuscript with input from all authors. All authors contributed to the manuscript and approved the final version.

DECLARATION OF INTERESTS

P.X. holds the position of Chief Technology Officer (CTO) at Airy Technologies. He declares that there are no additional financial or personal interests that could be perceived as a conflict of interest in relation to the research presented in this paper. P.X., R.C., and Y.L. are the authors of a relevant patent (202410853167.6).

SUPPLEMENTAL INFORMATION

It can be found online at https://doi.org/10.1016/j.xinn.2024.100757.

LEAD CONTACT WEBSITE

The lead contact website is www.xipenglab.com.

The Innovation, Volume *6*

Supplemental Information

Fast reconstruction and optical-sectioning three-dimensional structured illumination microscopy

Ruijie Cao, Yaning Li, Wenyi Wang, Yunzhe Fu, Xiaoyu Bu, Dilizhatai Saimi, Jing Sun, Xichuan Ge, Shan Jiang, Yuru Pei, Baoxiang Gao, Zhixing Chen, Meiqi Li, and Peng Xi

Supplementary material of "Fast-reconstruction and optical-sectioning three-dimensional structured illumination microscopy"

Ruijie Cao[1,2], Yaning Li[1,2,3], Wenyi Wang[1,2,4], Yunzhe Fu[1,2], Xiaoyu Bu[5], Dilizhatai Saimi[6], Jing Sun[5], Xichuan Ge[4], Shan Jiang[7], Yuru Pei[8], Baoxiang Gao[4], Zhixing Chen[6,9], Meiqi Li[10,*], Peng Xi[1,2,*]

[1]Department of Biomedical Engineering, College of Future Technology, Peking University, Beijing 100871, China.

[2]National Biomedical Imaging Center, College of Future Technology, Peking University, Beijing 100871, China.

[3]China Academy of Space Technology， Beijing Institute of Space Mechanics and Electricity, 100094.

[4]Airy Technologies Co., Ltd., Beijing 100081, China.

[5]Key Laboratory of Analytical Science and Technology of Hebei Province, College of Chemistry and Materials Science, Hebei University, Baoding 071002, China.

[6]College of Future Technology, Institute of Molecular Medicine, National Biomedical Imaging Center, Beijing Key Laboratory of Cardiometabolic Molecular Medicine, Peking University, Beijing 100871, China.

[7]Institute of Biomedical Engineering, Beijing Institute of Collaborative Innovation, Beijing, China.

[8]Key Laboratory of Machine Perception (MOE), Department of Machine Intelligence, Peking University, Beijing 100871, China.

[9]Peking-Tsinghua Center for Life Science, Academy for Advanced Interdisciplinary Studies, Peking University, Beijing 100871, China.

[10]School of Life Sciences, Peking University, Beijing 100871, China.

*Correspondence： limeiqi@pku.edu.cn (M.L.), xipeng@pku.edu.cn (P.X.)

Table of Contents

Note S1. Reconstruction of traditional 3DSIM 2
Note S2. Principle of FO-3DSIM 4
Note S3. The relationship between traditional 3DSIM and FO-3DSIM 7
Supplementary Figures 10
Supplementary Movies 16
Supplementary Tables 18

Note S1. Reconstruction of traditional 3DSIM

Traditional 3DSIM reconstructions are based on the Wiener-3DSIM flowchart. The illumination distribution $I_{\theta,\varphi}(r,z)$ of 3DSIM in phase φ and angle θ can expressed as the sum of zero, first, and second harmonics of cosine functions with certain weight:

$$I_{\theta,\varphi}(r,z) = I_0 \cdot [1 + a_1(z) \cdot \cos(\omega r + \varphi) + a_2 \cdot \cos(2\omega r + 2\varphi)] \tag{1-1}$$

Where I_0 is the illumination intensity, $a_1(z) = a_1 \cos(2\pi\omega_z z)$, a_1 and a_2 are the modulation depths of the first and second harmonics, ω and ω_z are the spatial frequency in the *xoy* and *xoz* plane, (r,z) is the position parameter. Then, the emission distribution $D_{\theta,\varphi}(r,z)$ of sample $S(r,z)$ can be expressed as:

$$D_{\theta,\varphi}(r,z) = \left[S(r,z) \cdot I_{\theta,\varphi}(r,z)\right] * H(r,z) \tag{1-2}$$

Where $S(r,z)$ is the sample distribution, $H(r,z)$ is the point spread function, $*$ is the convolution function. Further, its 3D frequency domain expression can be given by:

$$\begin{aligned} D_{\theta,\varphi}(k_r,k_z) &= \left[S(k_r,k_z) \otimes I_{\theta,\varphi}(k_r,k_z)\right] \cdot H(r,z) \\ &= I_0 \begin{Bmatrix} O(k_r,k_z) + a_1(k_z) \cdot \left[S(k_r \pm \omega, k_z - \omega_z)e^{j\varphi} + S(k_r \pm \omega, k_z + \omega_z)e^{-j\varphi})e^{-j\varphi}\right] \\ +a_2 \cdot \left[S(k_{x,y} - 2\omega, k_z)e^{j2\varphi} + S(k_{x,y} + 2\omega, k_z)e^{-j2\varphi}\right] \end{Bmatrix} \cdot H(k_{x,y},k_z) \end{aligned} \tag{1-3}$$

In each angle of the illumination pattern, five even phases should be used to resolve the five components $S(k_r,k_z)$, $S(k_r \pm \omega, k_z - \omega_z) + S(k_r \pm \omega, k_z + p_z)$ and $S(k_r \pm 2\omega, \omega_z)$. Therefore, five equations can be listed in the format of the matrix:

$$\begin{bmatrix} D_{\theta,\varphi_1}(k) \\ D_{\theta,\varphi_2}(k) \\ D_{\theta,\varphi_3}(k) \\ D_{\theta,\varphi_4}(k) \\ D_{\theta,\varphi_5}(k) \end{bmatrix} = I_0 A \begin{bmatrix} S(k_r,k_z) \cdot H(k_{x,y},k_z) \\ [S(k_r - \omega, k_z - p_z) + S(k_r - \omega, k_z + p_z)] \cdot H(k_r,k_z) \\ [S(k_r + \omega, k_z - p_z) + S(k_r + \omega, k_z + p_z)] \cdot H(k_r,k_z) \\ S(k_r - 2\omega, k_z) \cdot H(k_r,k_z) \\ S(k_r + 2\omega, k_z) \cdot H(k_r,k_z) \end{bmatrix} \tag{1-4}$$

Where $A = \begin{bmatrix} a_0 & a_1 e^{j\varphi_1} & a_1 e^{-j\varphi_1} & a_2 e^{j2\varphi_1} & a_2 e^{-j2\varphi_1} \\ a_0 & a_1 e^{j\varphi_2} & a_1 e^{-j\varphi_2} & a_2 e^{j2\varphi_2} & a_2 e^{-j2\varphi_2} \\ a_0 & a_1 e^{j\varphi_3} & a_1 e^{-j\varphi_3} & a_2 e^{j2\varphi_3} & a_2 e^{-j2\varphi_3} \\ a_0 & a_1 e^{j\varphi_4} & a_1 e^{-j\varphi_4} & a_2 e^{j2\varphi_4} & a_2 e^{-j2\varphi_4} \\ a_0 & a_1 e^{j\varphi_5} & a_1 e^{-j\varphi_5} & a_2 e^{j2\varphi_5} & a_2 e^{-j2\varphi_5} \end{bmatrix}$, and the five frequency components $C_{0,\pm1,\pm2}(k_r,k_z)$ can be expressed as:

$$\begin{bmatrix} C_0(k_r,k_z) \\ C_{-1}(k_r,k_z) \\ C_{+1}(k_r,k_z) \\ C_{-2}(k_r,k_z) \\ C_{+2}(k_r,k_z) \end{bmatrix} = \begin{bmatrix} S(k_r,k_z) \cdot H(k_{x,y},k_z) \\ \left[S(k_r - p_{x,y}, k_z - p_z) + S(k_r - p_{x,y}, k_z + p_z)\right] \cdot H(k_r,k_z) \\ \left[S(k_r + p_{x,y}, k_z - p_z) + S(k_r + p_{x,y}, k_z + p_z)\right] \cdot H(k_r,k_z) \\ S(k_r - 2p_{x,y}, k_z) \cdot H(k_r,k_z) \\ S(k_r + 2p_{x,y}, k_z) \cdot H(k_r,k_z) \end{bmatrix} = \frac{1}{I_0} A^{-1} \begin{bmatrix} D_{\theta,\varphi_0}(k_r,k_z) \\ D_{\theta,\varphi_1}(k_r,k_z) \\ D_{\theta,\varphi_2}(k_r,k_z) \\ D_{\theta,\varphi_3}(k_r,k_z) \\ D_{\theta,\varphi_4}(k_r,k_z) \end{bmatrix} \tag{1-5}$$

Where $[\cdot]^{-1}$ is the symbol of the matrix inverse transform. Then five frequency components $\boldsymbol{C}_n(k_r,k_z)$ are

shifted to the correct position $C_{ns}(k_r, k_z)$ laterally. $n = 0, \ \pm 1, \pm 2$ represents the index of five frequency components.

$$C_{ns}(k_r, k_z) = C_n(k_r, k_z)\ , n = 0$$
$$C_{ns}(k_r, k_z) = F\{F^{-1}[C_n(k_r, k_z)] \cdot e^{-jn2\pi p_{x,y}}\}\ , n = -1, -2$$
$$C_{ns}(k_r, k_z) = F\{F^{-1}[C_n(k_r, k_z)] \cdot e^{jn2\pi p_{x,y}}\}\ , n = +1, +2$$

(1-6)

Then, the super-resolution image can be expressed as:

$$C_{SR}(k_r, k_z) = \sum_{n=-2}^{2} C_{ns}(k_r, k_z) \tag{1-7}$$

The detailed spectrum optimization including notch-filter, Weiner filter, or two-step filter is dismissed here, as seen in previous work. In the process of traditional reconstruction. The most time-consuming steps are the frequency separation and components shift, where the 3D matrix is involved to conduct the Fourier and inverse Fourier transform. Moreover, the 3D Fourier transform and its optimization steps usually need more than 6 layers in the step of frequency shift to guarantee correctness. Fewer layers to conduct frequency operation may change the final results in the spatial domain, making it difficult to reconstruct fewer *z*-layers.

Note S2. Principle of FO-3DSIM

Traditional 3DSIM reconstruction is based on frequency separation. Here, we develop FO-3DSIM based on spatial reconstruction. According to equation (1-2), when the illumination pattern has a phase shift r_i in *xoy* plane, the emission distribution $D_{\theta,\varphi}(r,z)$ can be further expressed as:

$$D_{\theta,\varphi}(r,z) \ = S(r,z) \cdot I_\theta(r' - r_i, z) * H(r,z) \tag{2-1}$$

Where r' is the corrected position parameter. Due to the process of SIM being linear, we can assume there exist coefficients $o_{\theta,i}(r,z)$, *i* = 1, 2, 3, 4, 5, that makes the following equation always true for any $O(r,z)$ and $H(r,z)$:

$$SR_{0,\theta}(r,z) = \sum_{i=1}^{5} \ o_{\theta,i}(r,z) \cdot D_{\theta,\varphi}(r,z) \tag{2-2}$$

Where $SR_{0,\theta}(r,z)$ denotes the initial super-resolution image in angle θ. And effective PSF can be obtained by multiplying the wide-filed PSF $H(r,z)$ with a modulation function $1 + \cos(\omega_z z) \cdot \cos(\omega r) + \cos(2\omega r)$. So, $SR_{0,\theta}(r,z)$ can also written as:

$$SR_{0,\theta}(r,z) = S(r,z) * \{[1 + \cos(\omega_z z) \cdot \cos(\omega r) + \cos(2\omega r)] \cdot H(r,z)\} \tag{2-3}$$

Let equation (2-2) equals to equation (2-3). And the illumination pattern can be expressed as: $I_\theta(r' - r_i, z) = I_0 \cdot [1 + a_1 \cdot \cos(\omega_z z) \cdot \cos(\omega r' + \varphi - \omega r_i) + a_2 \cdot \cos\,(2\omega r' + 2\varphi - 2\omega r_i)]$. We can get the following equation:

$$\begin{aligned} & I_0 \sum_{i=1}^{5} e_{\theta,i}(r,z) \cdot [1 + a_1 \cdot \cos(\omega_z z) \cdot \cos(\omega r' + \varphi - \omega r_i) + a_2 \cdot \cos(2\omega r' + 2\varphi - 2\omega r_i)] \\ & = 1 + \cos(\omega_z z) \cdot \cos(\omega r - \omega r') + \cos(2\omega r - 2\omega r') \\ & = 1 + \cos(\omega_z z) \cdot \cos[(\omega r + \varphi) - (\omega r' + \varphi)] + \cos[2(\omega r + \varphi) - 2(\omega r' + \varphi)] \end{aligned} \tag{2-4}$$

We rewrite both side of equation (2-4) as functions of $\cos(\omega r' + \varphi)$ and $\sin(\omega r' + \varphi)$, equation (2-4) can be written as:

$$\begin{aligned} & I_0 \sum_{i=1}^{5} o_{\theta,i}(r,z) + a_1 \cos(\omega_z z) \cdot I_0 \sum_{i=1}^{5} o_{\theta,i}(r,z) \cdot [\cos(\omega r' + \varphi) \cos(\omega r_i) + \sin(\omega r' + \varphi) \cdot \sin(\omega r_i)] + \\ & a_2 I_0 \sum_{i=1}^{5} o_{\theta,i}(r,z) \cdot [\cos(2\omega r' + 2\varphi) \cdot \cos(2\omega r_i) + \sin(2\omega r' + 2\varphi) \cdot \sin(2\omega r_i)] \\ & = 1 + \cos(\omega_z z) \cdot [\cos(\omega r + \varphi) \cdot \cos(\omega r' + \varphi) + \sin(\omega r + \varphi) \cdot \sin(\omega r' + \varphi)] \\ & + \cos(2\omega r + 2\varphi) \cdot \cos(2\omega r' + 2\varphi) + \sin(2\omega r + 2\varphi) \cdot \sin(2\omega r' + 2\varphi) \end{aligned} \tag{2-5}$$

Considering the orthogonality of $\cos(\omega r' + \varphi)$ and $\sin(\omega r' + \varphi)$, we can get the following equations:

$$I_0 \sum_{i=1}^{5} o_{\theta,1}(r,z) = 1$$

$$a_1 I_0 \sum_{i=1}^{5} o_{\theta,2}(r,z) \cdot \cos\,(\omega r_i) = \cos(\omega r + \varphi)$$

$$a_1 I_0 \sum_{i=1}^{5} o_{\theta,3}(r,z) \cdot \sin\,(\omega r_i) = \sin(\omega r + \varphi)$$

$$a_2 I_0 \sum_{i=1}^{5} o_{\theta,4}(r,z) \cdot \cos(2\omega r_i) = \cos(2\omega r + 2\varphi)$$

$$a_2 I_0 \sum_{i=1}^{5} o_{\theta,5}(r,z) \cdot \sin(2\omega r_i) = \sin(2\omega r + 2\varphi) \tag{2-6}$$

To resolve the coefficients $c_{\theta,i}(r,z)$, we assume the phase shift ωr_i is $0, \frac{2}{5}\pi, \frac{4}{5}\pi, \frac{6}{5}\pi, \frac{8}{5}\pi$. And the following linear equation should be resolved:

$$\begin{bmatrix} o_{\theta,1}(r,z) \\ o_{\theta,2}(r,z) \\ o_{\theta,3}(r,z) \\ o_{\theta,4}(r,z) \\ o_{\theta,5}(r,z) \end{bmatrix} \begin{bmatrix} 1 & 1 & 1 & 1 & 1 \\ \cos 0 & \cos\frac{2}{5}\pi & \cos\frac{4}{5}\pi & \cos\frac{6}{5}\pi & \cos\frac{8}{5}\pi \\ \sin 0 & \sin\frac{2}{5}\pi & \sin\frac{4}{5}\pi & \sin\frac{6}{5}\pi & \sin\frac{8}{5}\pi \\ \cos 0 & \cos\frac{4}{5}\pi & \cos\frac{8}{5}\pi & \cos\frac{12}{5}\pi & \cos\frac{16}{5}\pi \\ \sin 0 & \sin\frac{4}{5}\pi & \sin\frac{8}{5}\pi & \sin\frac{12}{5}\pi & \sin\frac{16}{5}\pi \end{bmatrix} = \begin{bmatrix} \frac{1}{I_0} \\ \frac{\cos(\omega r+\varphi)}{a_1 I_0} \\ \frac{\sin(\omega r+\varphi)}{a_1 I_0} \\ \frac{\cos(2\omega r+2\varphi)}{a_2 I_0} \\ \frac{\sin(2\omega r+2\varphi)}{a_2 I_0} \end{bmatrix} \tag{2-7}$$

Because 3DSIM is suitable for thick sample imaging, we subtract the wide-field image in this step and add the optical-sectioning results in the following steps. Equation (2-7) can be rewritten as:

$$\begin{bmatrix} o_{\theta,1}(r,z) \\ o_{\theta,2}(r,z) \\ o_{\theta,3}(r,z) \\ o_{\theta,4}(r,z) \\ o_{\theta,5}(r,z) \end{bmatrix} \begin{bmatrix} 0 & 0 & 0 & 0 & 0 \\ \cos 0 & \cos\frac{2}{5}\pi & \cos\frac{4}{5}\pi & \cos\frac{6}{5}\pi & \cos\frac{8}{5}\pi \\ \sin 0 & \sin\frac{2}{5}\pi & \sin\frac{4}{5}\pi & \sin\frac{6}{5}\pi & \sin\frac{8}{5}\pi \\ \cos 0 & \cos\frac{4}{5}\pi & \cos\frac{8}{5}\pi & \cos\frac{12}{5}\pi & \cos\frac{16}{5}\pi \\ \sin 0 & \sin\frac{4}{5}\pi & \sin\frac{8}{5}\pi & \sin\frac{12}{5}\pi & \sin\frac{16}{5}\pi \end{bmatrix} = \begin{bmatrix} \frac{1}{I_0} \\ \frac{\cos(\omega r+\varphi)}{a_1 I_0} \\ \frac{\sin(\omega r+\varphi)}{a_1 I_0} \\ \frac{\cos(2\omega r+2\varphi)}{a_2 I_0} \\ \frac{\sin(2\omega r+2\varphi)}{a_2 I_0} \end{bmatrix} \tag{2-8}$$

It can be seen that coefficients $o_{\theta,i}(r,z)$ have no relevance of *z*, so they only depend on the lateral parameter *r*. So, the super-resolution image $SR_{0,-}(r,z)$ (minus the 0th component) can be expressed as:

$$SR_{0,-}(r,z) = \sum_{\theta=1}^{3} \sum_{i=1}^{5} o_{\theta,i}(r,z) \cdot D_{\theta,i}(r,z) \tag{2-9}$$

Then, to reduce the honeycomb artifacts, we use a notch filter to suppress the high-frequency peaks. The notch filter $notch(x,y,z,n)$ is the same form as Open-3DSIM, but it did not notch the center frequency component because the minus of wide-field image. The notched frequency domain of super-resolution image can be expressed as:

$$F_{SR_{0,-}}(r,z) = \left[\prod_{n=-2,-1,1,2} notch(x,y,z,n)\right] \cdot F[SR_{0,-}(r,z)] \tag{2-10}$$

Where $notch(x,y,z,0) = 1 - d \cdot exp\left[\left(\frac{x^2+y^2}{|p_{x,y}|^2} + \frac{z^2}{|p_z|^2}\right)/2/w^2\right]$ is the notch-filter designed according to the estimated frequency vector on the *xoy* and *yoz* plane, $notch(x,y,z,n)$ is the corresponding shifted notch filter in frequency-domain position on the base of $notch(x,y,z,0)$, d and w are the notch depth and width, respectively, $F[\cdot]$ is the symbol of Fourier transform,$\prod[\cdot]$ is the multiplicative multiplication symbol.

Then, we use a two-step spectrum filter of $Filter1(k) = \frac{Apo}{OTF_{notch}+w_1^2}$ and $Filter2(k) = \frac{Apo}{OTF_{notch}+w_2^2}$ similar to Open-3DSIM to minimize the artifacts of reconstruction, where $OTF_{notch} = \sum_{n=-2,-1,1,2} m(n) \cdot OTF(x,y,z,n) \cdot$

$notch(x, y, z, n)$, $m(n)$ is the weight coefficient of different Fourier orders, $OTF(x, y, z, n)$ is the leaky-cone-shaped optical transfer function (OTF) shifted to the n-th place in the frequency domain, w_1 and w_2 are the parameters to adjust the filters. The filtered super-resolution image $SR_1(r, z)$ can be expressed as:

$$SR_1(r, z) = F^{-1}\left[Filter1(k) \cdot Filter2(k) \cdot F_{SR_{0,-}}(r, z)\right] \quad (2\text{-}11)$$

Previously, we subtracted the wide-filed image to suppress the defocused background. Here, we use the HiLo algorithm to remove the defocused background. HiLo microscopy uses one image with low spectrum spread patterned illumination and one wide-field image to get a background removed image. 3DSIM has lower harmonics (~1.5 spread spectrum) compared with 2DSIM, so introducing HiLo into 3DSIM corresponds to the requirement of HiLo. Assuming the raw patterned image with angle θ and i-th phase is $D_{\theta,i}(r, z)$. The HiLo image $OS(r, z)$ can be expressed as:

$$OS(r, z) = \sum_{\theta=1}^{3}\left\{Lo\left[D_{\theta,1}(r, z)\right] + Hi\left[\sum_{i=1}^{5} D_{\theta,i}(r, z)\,/5\right]\right\}/3 \quad (2\text{-}12)$$

Where $Lo[\cdot]$ and $Hi[\cdot]$ are the low-pass and high-pass filter, respectively, and we average the three angles of illumination to avoid light intensity difference in three angles. At last, the final super-resolution image $SR_2(r, z)$ can be expressed as:

$$SR_2(r, z) = SR_1(r, z) + OS(r, z) \quad (2\text{-}13)$$

Note S3. The relationship between traditional 3DSIM and FO-3DSIM

Here, we will discuss that the principle of FO-3DSIM is identical to traditional 3DSIM. The spatial reconstruction will not sacrifice the fidelity and resolution of traditional 3DSIM. We first rewrite equation (2-7) into the following equation:

$$\begin{bmatrix} o_1(r,z) \\ o_2(r,z) \\ o_3(r,z) \\ o_4(r,z) \\ o_5(r,z) \end{bmatrix} = \begin{bmatrix} O_{11} & O_{12} & O_{13} & O_{14} & O_{15} \\ O_{21} & O_{22} & O_{23} & O_{24} & O_{25} \\ O_{31} & O_{32} & O_{33} & O_{34} & O_{35} \\ O_{41} & O_{42} & O_{43} & O_{44} & O_{45} \\ O_{51} & O_{52} & O_{53} & O_{54} & O_{55} \end{bmatrix} \begin{bmatrix} \frac{1}{I_0} \\ \frac{\cos(\omega r+\varphi)}{a_1 I_0} \\ \frac{\sin(\omega r+\varphi)}{a_1 I_0} \\ \frac{\cos(2\omega r+2\varphi)}{a_2 I_0} \\ \frac{\sin(2\omega r+2\varphi)}{a_2 I_0} \end{bmatrix} \tag{3-1}$$

Where $\begin{bmatrix} O_{11} & O_{12} & O_{13} & O_{14} & O_{15} \\ O_{21} & O_{22} & O_{23} & O_{24} & O_{25} \\ O_{31} & O_{32} & O_{33} & O_{34} & O_{35} \\ O_{41} & O_{42} & O_{43} & O_{44} & O_{45} \\ O_{51} & O_{52} & O_{53} & O_{54} & O_{55} \end{bmatrix} = \begin{bmatrix} 1 & 1 & 1 & 1 & 1 \\ \cos 0 & \cos\frac{2}{5}\pi & \cos\frac{4}{5}\pi & \cos\frac{6}{5}\pi & \cos\frac{8}{5}\pi \\ \sin 0 & \sin\frac{2}{5}\pi & \sin\frac{4}{5}\pi & \sin\frac{6}{5}\pi & \sin\frac{8}{5}\pi \\ \cos 0 & \cos\frac{4}{5}\pi & \cos\frac{8}{5}\pi & \cos\frac{12}{5}\pi & \cos\frac{16}{5}\pi \\ \sin 0 & \sin\frac{4}{5}\pi & \sin\frac{8}{5}\pi & \sin\frac{12}{5}\pi & \sin\frac{16}{5}\pi \end{bmatrix}^{-1}$ denotes the intermediate matrix.

According to the equation (2-10), the super-resolution image $SR_0(r,z)$ can be further expressed as:

$$\begin{aligned} SR_0(r,z) &= \textstyle\sum_{i=1}^{5} o_i(r,z) \cdot D_i(r,z) \\ &= \frac{1}{I_0}\textstyle\sum_{i=1}^{5} O_{i1} D_i(r,z) + \frac{1}{a_1 I_0}\sum_{i=1}^{5} O_{i2} D_i(r,z) \cdot \cos(\omega r+\varphi) + \frac{1}{a_1 I_0}\sum_{i=1}^{5} O_{i3} D_i(r,z) \cdot \sin(\omega r+\varphi) \\ &\quad + \frac{1}{a_2 I_0}\textstyle\sum_{i=1}^{5} O_{i4} D_i(r,z) \cdot \cos(2\omega r+2\varphi) + \frac{1}{a_2 I_0}\sum_{i=1}^{5} O_{i5} D_i(r,z) \cdot \sin(2\omega r+2\varphi) \end{aligned} \tag{3-2}$$

To simplify the equation above, we further define $E_i(r,z)$ as:

$$\begin{aligned} E_0(r,z) &= \frac{1}{I_0}\textstyle\sum_{i=1}^{5} O_{i1} D_i(r,z) \\ E_1(r,z) &= \frac{1}{a_1 I_0}\textstyle\sum_{i=1}^{5} O_{i2} D_i(r,z) \\ E_2(r,z) &= \frac{1}{a_1 I_0}\textstyle\sum_{i=1}^{5} O_{i3} D_i(r,z) \\ E_3(r,z) &= \frac{1}{a_2 I_0}\textstyle\sum_{i=1}^{5} O_{i4} D_i(r,z) \\ E_4(r,z) &= \frac{1}{a_2 I_0}\textstyle\sum_{i=1}^{5} O_{i5} D_i(r,z) \end{aligned} \tag{3-3}$$

Without loss of generality, let $I_0 = 1$, the super-resolution image $SR_0(r,z)$ can be simplified as:

$$\begin{aligned} SR_0(r,z) &= E_0(r,z) + E_1(r,z)\cos(\omega r+\varphi) + E_2(r,z)\sin(\omega r+\varphi) + E_3(r,z)\cos(2\omega r+2\varphi) \\ &\quad + E_4(r,z)\sin(2\omega r+2\varphi) \end{aligned}$$

(3-4)

Then, equation (2-3) can be expressed as $D_i(r,z) = S(r,z) \cdot [1 + \cos(\omega r + \varphi_i) + \cos(2\omega r + 2\varphi_i)] * H(r,z)$, $R_i(r,z)$ can be expressed as:

$$E_0(r,z) = S(r,z) * H(r,z)$$

$$E_1(r,z) = [S(r,z) \cdot \cos(\omega r + \varphi)] * H(r,z)$$

$$E_2(r,z) = [S(r,z) \cdot \sin(\omega r + \varphi)] * H(r,z)$$

$$E_4(r,z) = [S(r,z) \cdot \cos(2\omega r + 2\varphi)] * H(r,z)$$

$$E_5(r,z) = [S(r,z) \cdot \sin(2\omega r + 2\varphi)] * H(r,z) \quad (3\text{-}5)$$

According to the equation that $\cos(\omega r + \varphi) = \frac{e^{j(\omega r+\varphi)} + e^{-j(\omega r+\varphi)}}{2}$, $E_1(r,z)$ can be additionally expressed as:

$$E_1(r,z) = \left[S(r,z) \cdot \frac{e^{j(\omega r+\varphi)} - e^{-j(\omega r+\varphi)}}{2}\right] * H(r,z)$$

$$= \frac{e^{j\varphi}}{2}\left[S(r,z)e^{j\omega r}\right] * H(r,z) + \frac{e^{-j\varphi}}{2}\left[S(r,z)e^{-j\omega r}\right] * H(r,z)$$

$$= \frac{e^{j\varphi}}{2} F^{-1}[S(k-\omega,z) \cdot H(k,z)] + \frac{e^{-j\varphi}}{2} F^{-1}[S(k+\omega,z) \cdot H(k,z)]$$

(3-6)

Similarly, $E_2(r,z)$, $E_3(r,z)$, $E_4(r,z)$ can be expressed as:

$$E_2(r,z) = \frac{e^{j\varphi}}{2j} F^{-1}[S(k-\omega,z) \cdot H(k,z)] - \frac{e^{-j\varphi}}{2j} F^{-1}[S(k+\omega,z) \cdot H(k,z)]$$

$$E_3(r,z) = \frac{e^{j2\varphi}}{2} F^{-1}[S(k-2\omega,z) \cdot H(k,z)] + \frac{e^{-j2\varphi}}{2} F^{-1}[S(k+2\omega,z) \cdot H(k,z)]$$

$$E_4(r,z) = \frac{e^{j2\varphi}}{2j} F^{-1}[S(k-2\omega,z) \cdot H(k,z)] - \frac{e^{-j2\varphi}}{2j} F^{-1}[S(k+2\omega,z) \cdot H(k,z)] \quad (3\text{-}7)$$

After solving the linear formula of equation (3-6) and (3-7), $S(k \pm \omega, z) \cdot H(k,z)$ and $S(k \pm 2\omega, z) \cdot H(k,z)$ can be resolved:

$$S(k-\omega,z) \cdot H(k,z) = e^{-j\varphi} \cdot F[E_1(r,z) + jE_2(r,z)]$$

$$S(k+\omega,z) \cdot H(k,z) = e^{j\varphi} \cdot F[E_1(r,z) - jE_2(r,z)]$$

$$S(k-2\omega,z) \cdot H(k,z) = e^{-j2\varphi} \cdot F[E_3(r,z) + jE_4(r,z)]$$

$$S(k+2\omega,z) \cdot H(k,z) = e^{j2\varphi} \cdot F[E_3(r,z) + jE_4(r,z)]$$

(3-8)

In traditional 3DSIM reconstruction, the frequency components $M_0(k,z)$, $M_{-1}(k,z)$ can be expressed as:

$$M_0(k,z) = S(k,z) \cdot H(k,z)$$

$$M_{-1}(k,z) = \frac{1}{2} S(k,z) \cdot H(k-\omega,z)$$

$$= \frac{1}{2} F\left\{F^{-1}[S(k+\omega, z) \cdot H(k,z)] e^{j\omega r}\right\}$$

$$= \frac{1}{2} F\left\{F^{-1}\left[e^{j\varphi} \cdot F[E_1(r,z) - jE_2(r,z)]\right] e^{j\omega r}\right\}$$

$$= \frac{1}{2} F\left\{[E_1(r,z) - jE_2(r,z)] e^{j(\omega r+\varphi)}\right\}$$

(3-9)

Similarly, $M_{+1}(k,z)$, $M_{-2}(k,z)$, and $M_{+2}(k,z)$ can be expressed as:

$$M_{+1}(k,z) = \frac{1}{2} F\left\{[E_1(r,z) + jE_2(r,z)] e^{-j(\omega r+\varphi)}\right\}$$

$$M_{-2}(k,z) = \frac{1}{2} F\left\{[E_3(r,z) - jE_4(r,z)] e^{j2(\omega r+\varphi)}\right\}$$

$$M_{+2}(k,z) = \frac{1}{2} F\left\{[E_3(r,z) + jE_4(r,z)] e^{-j2(\omega r+\varphi)}\right\}$$

(3-10)

After considering equation (3-9) and (3-10), the super-resolution image can be expressed as the following equation in traditional 3DSIM reconstruction:

$$SR_0(r,z) = F^{-1}\{M_0(k,z) + M_{-1}(k,z) + M_{+1}(k,z) + M_{-2}(k,z) + M_{+2}(k,z)\}$$

$$= F^{-1}\left\{\begin{matrix} S(k,z) \cdot H(k,z) + \frac{1}{2} F\left\{[E_1(r,z) - jE_2(r,z)] e^{j(\omega r+\varphi)}\right\} + \frac{1}{2} F\left\{[E_1(r,z) + jE_2(r,z)] e^{-j(\omega r+\varphi)}\right\} \\ + \frac{1}{2} F\left\{[E_3(r,z) - jE_4(r,z)] e^{j2(\omega r+\varphi)}\right\} + \frac{1}{2} F\left\{[E_3(r,z) + jE_4(r,z)] e^{-j2(\omega r+\varphi)}\right\} \end{matrix}\right\}$$

$$= E_0(r,z) + E_1(r,z)\cos(\omega r + \varphi) + E_2(r,z)\sin(\omega r + \varphi) + E_3(r,z)\cos(2\omega r + 2\varphi) +$$

$$E_4(r,z)\sin(2\omega r + 2\varphi)$$

(3-11)

We can find that equation (3-11) is the same formulation as equation (3-4). As a result, FO-3DSIM is equal to traditional 3DSIM mathematically.

Supplementary Figures

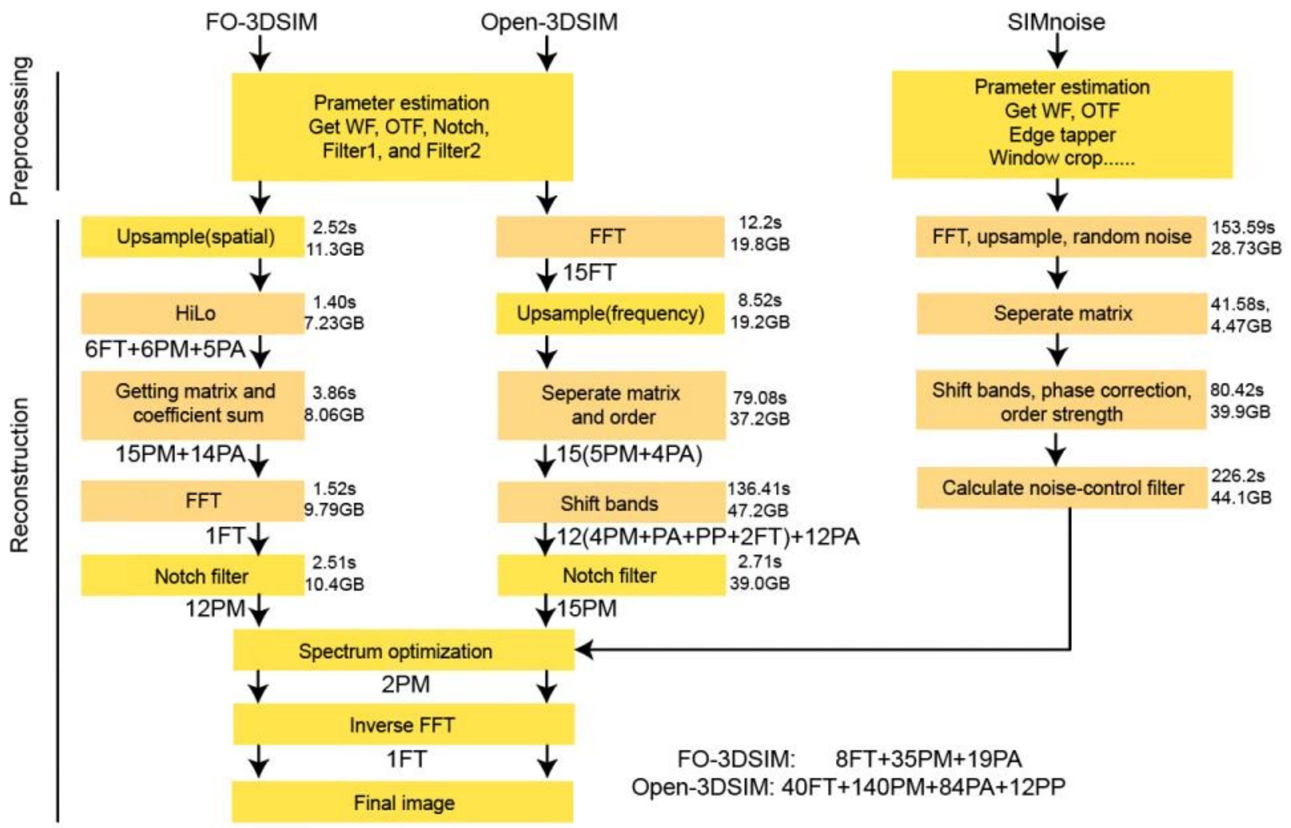

Figure S1. Comparison of reconstruction time of immediate steps in FO-3DSIM, Open-3DSIM, and SIMnoise. The image size is 512×512×41, Both FO-3DSIM, Open-3DSIM, and SIMnoise are written by CPU only for fair comparison. FT, (inverse) Fourier transforms, PM, pointwise multiplications, PA, pointwise multiplications additions, PP, pointwise power.

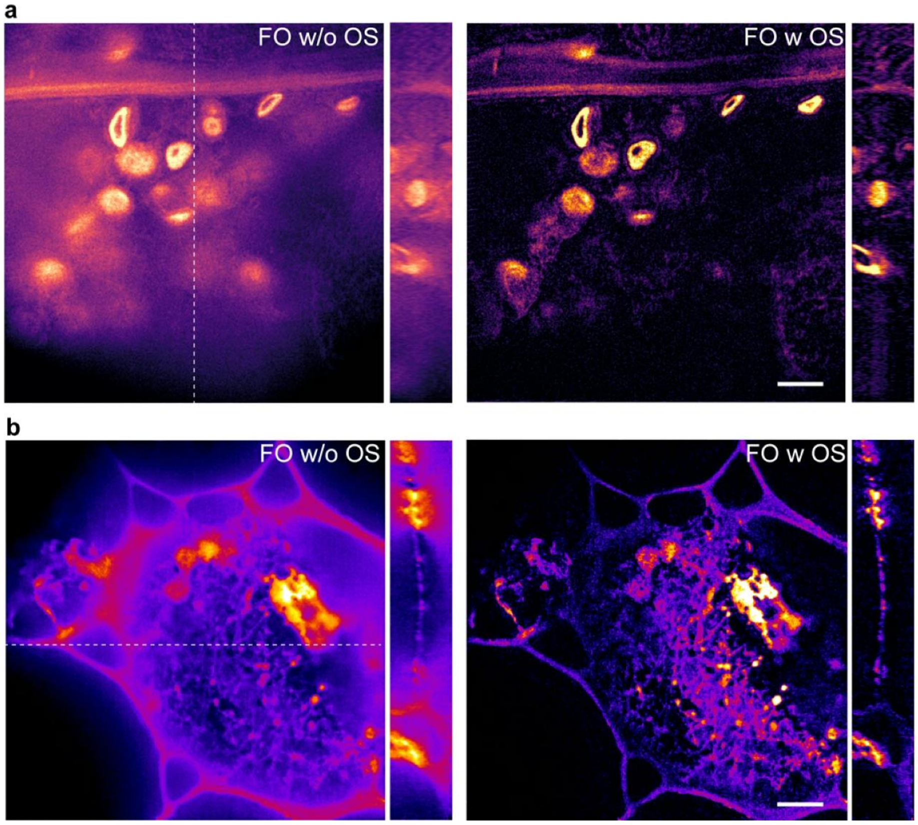

Figure S2. Effect of combining OS-SIM into FO-3DSIM. Comparison between FO-3DSIM with OS-SIM (FO w OS) and FO-3DSIM without OS-SIM (FO w/o OS). Scale bar: 4 μm.

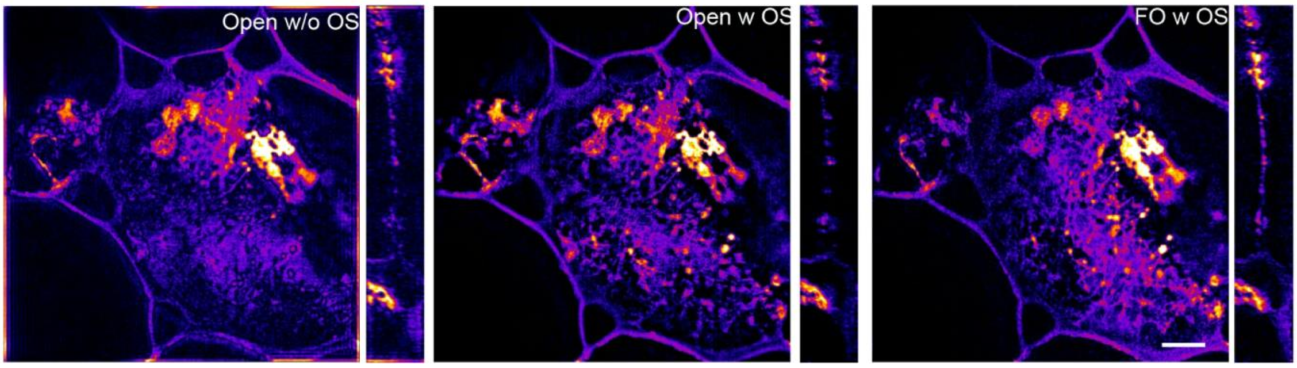

Figure S3. Traditional 3DSIM can also benefit from combining OS-SIM and 3DSIM. Scale bar: 4 μm. Axial scale: 44 layers, 125nm per layer.

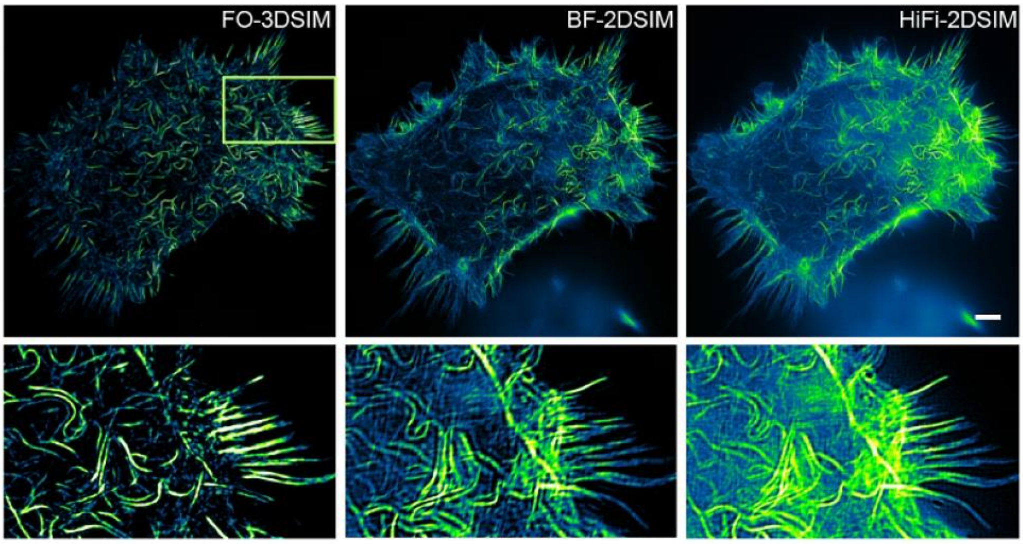

Figure S4. Comparison between FO-3DSIM and state-of-art 2DSIM results including BF-SIM and HiFi-SIM.

The data is from Fig.5(a) in the manuscript of live actin filament. Scale bar: 4 μm.

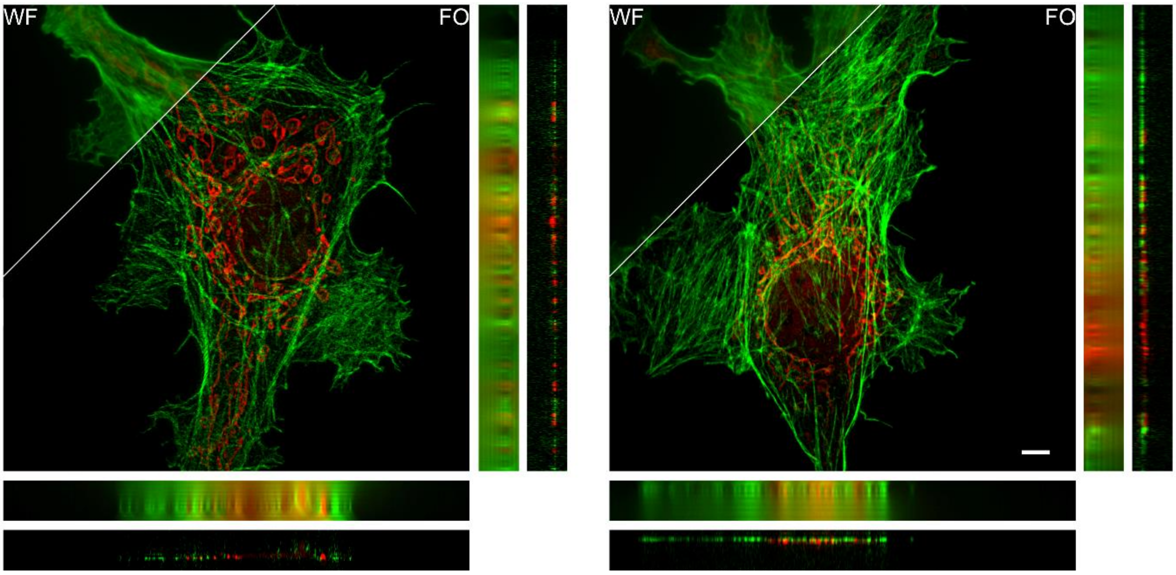

Figure S5. FO-3DSIM assists in multi-color imaging of actin filament and mitochondria. Scale bar: 4 μm.

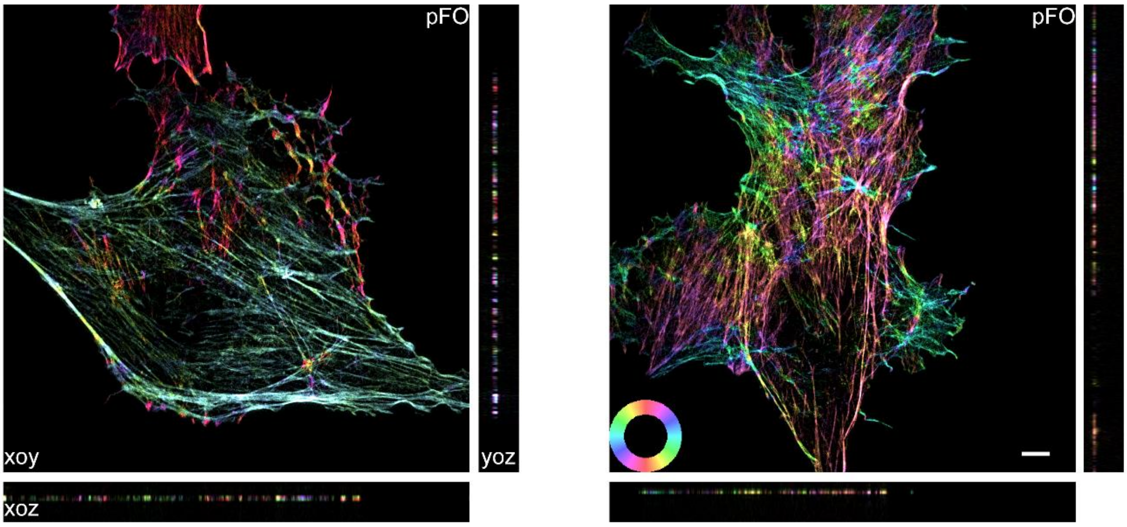

Figure S6. FO-3DSIM resolves the dipole orientation of actin filaments. Scale bar: 4 μm.

Supplementary Movies

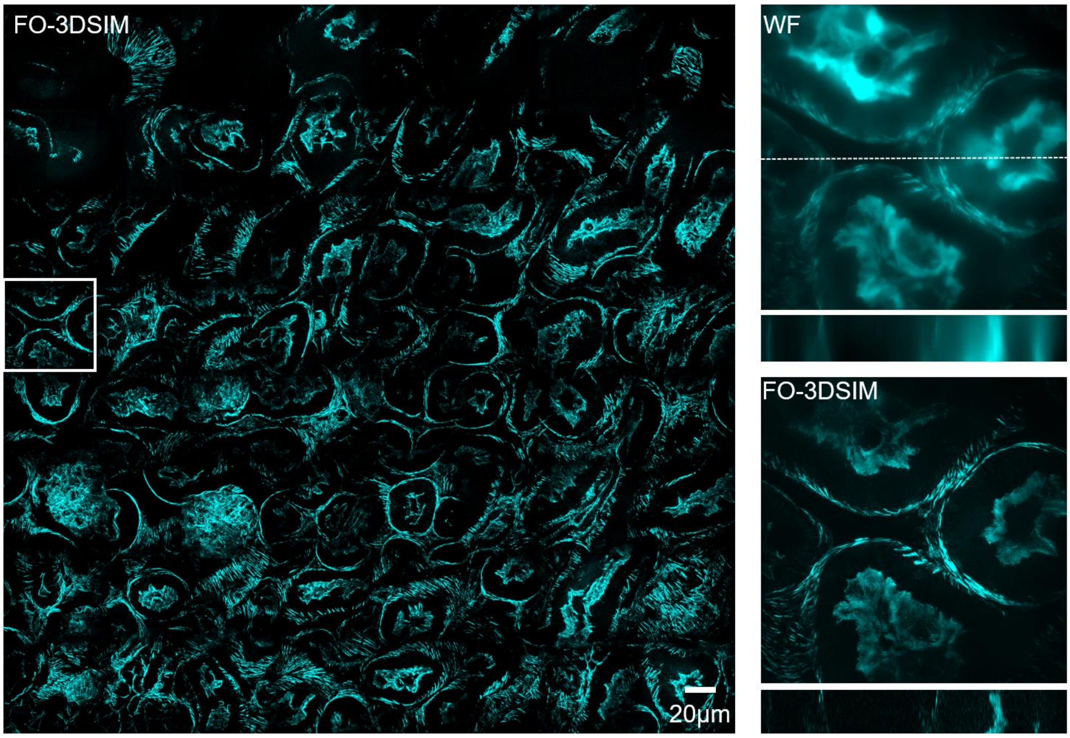

Movie S1. 3D Large-FOV super-resolution imaging.

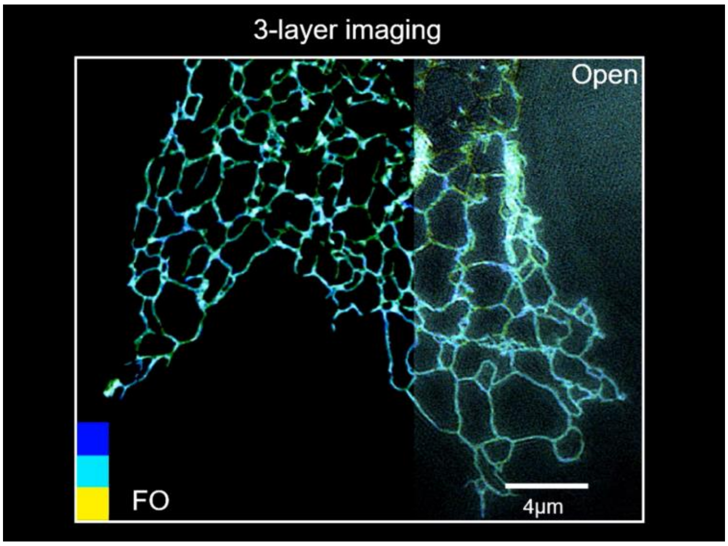

Movie S2. FO-3DSIM enables limited-layer reconstruction, and produces lower photodamage.

Supplementary Tables

Supplementary Table 1. Information of samples in this article

Figure	System	Sample	Excitation wavelength	Pixel size	NA
Fig. 1(a)	DMD-3DSIM	Black algal leaves	561nm	65nm	1.49
Fig. 2(e, f)	N-SIM2	Mouse kidney section	561nm	65nm	1.49
Fig. 3(a)	OMX	Actin	Open-source data from Open-3DSIM		
Fig. 3(c, d)	DMD-3DSIM	Black algal leaves	Open-source data from DMD-3DSIM		
Fig. 3(f)	DMD-3DSIM	Veins in oleander leaves	561nm	65nm	1.49
Fig. 3(g)	DMD-3DSIM	Mouse kidney section	561nm	65nm	1.49
Fig. 4	N-SIM2	Mouse kidney section	561nm	65nm	1.49
Fig. 5	OMX	Actin	561nm	80nm	1.40
Fig. 6	OMX	ER tubes	561nm	80nm	1.40
Fig. S2(a)	DMD-3DSIM	Black algal leaves	Open-source data from DMD-3DSIM		
Fig. S2(b)	DMD-3DSIM	Veins in oleander leaves	561nm	65nm	1.49
Fig. S3	DMD-3DSIM	Veins in oleander leaves	561nm	65nm	1.49
Fig. S4	OMX	Actin	561nm	80nm	1.40
Fig. S5	Polar-SIM	Actin + Mitochondria	561nm / 640nm	65nm	1.49
Fig. S6	Polar-SIM	Actin	561nm	65nm	1.49
